# N^6^-methyladenosine mRNA marking promotes selective translation of regulons required for human erythropoiesis

**DOI:** 10.1038/s41467-019-12518-6

**Published:** 2019-10-10

**Authors:** Daniel A. Kuppers, Sonali Arora, Yiting Lim, Andrea R. Lim, Lucas M. Carter, Philip D. Corrin, Christopher L. Plaisier, Ryan Basom, Jeffrey J. Delrow, Shiyan Wang, Housheng Hansen He, Beverly Torok-Storb, Andrew C. Hsieh, Patrick J. Paddison

**Affiliations:** 1 Human Biology Division, Fred Hutchinson Cancer Research Center, Seattle, WA 98109 USA; 2 Molecular and Cellular Biology Program, University of Washington, Seattle, WA 98195 USA; 3 School of Biological and Health Systems Engineering, Arizona State University, Tempe, AZ 85281 USA; 4 Genomics Shared Resource, Fred Hutchinson Cancer Research Center, Seattle, WA 98109 USA; 5 Princess Margaret Cancer Centre/University Health Network, Toronto, ON Canada; 6 Clinical Research Division, Fred Hutchinson Cancer Research Center, Seattle, WA 98109 USA; 7 School of Medicine, University of Washington, Seattle, WA 98195 USA

**Keywords:** Methylation analysis, Differentiation, RNA modification, Translation

## Abstract

Many of the regulatory features governing erythrocyte specification, maturation, and associated disorders remain enigmatic. To identify new regulators of erythropoiesis, we utilize a functional genomic screen for genes affecting expression of the erythroid marker CD235a/GYPA. Among validating hits are genes coding for the N^6^-methyladenosine (m^6^A) mRNA methyltransferase (MTase) complex, including, *METTL14*, *METTL3*, and *WTAP*. We demonstrate that m^6^A MTase activity promotes erythroid gene expression programs through selective translation of ~300 m^6^A marked mRNAs, including those coding for SETD histone methyltransferases, ribosomal components, and polyA RNA binding proteins. Remarkably, loss of m^6^A marks results in dramatic loss of H3K4me3 marks across key erythroid-specific KLF1 transcriptional targets (e.g., Heme biosynthesis genes). Further, each m^6^A MTase subunit and a subset of their mRNAs targets are required for human erythroid specification in primary bone-marrow derived progenitors. Thus, m^6^A mRNA marks promote the translation of a network of genes required for human erythropoiesis.

## Abstract (web summary)

Erythropoiesis can be regulated by transcriptional, epigenetic, and post-transcriptional mechanisms. Here the authors report that N^6^-methyladenosine mRNA methyltransferase complex stimulates erythropoiesis by promoting translation of specific mRNAs.

## Introduction

In humans, erythropoiesis occurs in a step-wise lineage progression from bi-potent MEP to mature erythrocytes which fulfill the daily requirement for ~2 × 10^11^ new erythrocytes^1^. The complex process of erythroid lineage commitment and maturation is governed by multiple regulatory mechanisms, including: (a) transcriptional, epigenetic, and RNAi-dependent promotion of lineage-specific cell characteristics and restriction of developmental potential^2,3^; (b) lineage-specific cytokine signaling that promote cell survival and expansion of committed progenitors^4^; and (c) a network of ribosome-associated proteins (e.g., *RPS19*), which when mutated can trigger life-threatening anemias (e.g., Diamond–Blackfan anemia (DBA)) and myeloproliferative disease by blocking erythroid maturation^5^. However, owing to the limitations of deriving and manipulating human hematopoietic stem and progenitor cells (HSPCs) and erythroid progenitors, experimental investigations of erythropoiesis have been limited in scope. As a result, many additional regulatory factors governing human erythropoiesis likely await discovery.

N6-methyladenosine (m^6^A) is an abundant modification of mRNA with an increasingly important role in normal cell physiology^6–10^ and disease^11–15^. The core m^6^A methyltransferase complex (MTase) consists of a trimeric complex containing two proteins with conserved MTase (MT-A70) domains, METTL3 and METTL14 (refs. ^16,17^), and an additional subunit, the Wilms’ tumor 1-associating protein (WTAP)^18,19^. In cell-based models, m^6^A has been shown to participate in numerous types of mRNA regulation, including pre-mRNA processing^17,20,21^, mRNA translation efficiency^22^, mRNA stability^16^, and miRNA biogenesis^23^. In the context of hematopoiesis, m^6^A has recently been found to regulate the expansion and self-renewal of hematopoietic stem cells^24,25^, as well as functioning as a negative regulator of myelopoiesis and a potential driver of acute myeloid leukemia (AML)^11,26^.

In this study, we utilize a comprehensive CRISRP–Cas9-based screening approach to identify an essential regulatory role for m^6^A RNA methylation during erythropoiesis^27^. Loss of m^6^A through inhibition of the methyltransferase complex results in disruption of the erythroid transcriptional program without direct inhibition of previously identified master transcriptional regulators (e.g., GATA1, KLF1). Instead, we observe translational down regulation of a variety of genes with known or suspected roles in erythropoiesis, erythroid-related diseases, and/or hematopoietic progenitor cell function. In addition, maintenance of the erythroid transcriptional program is in part driven by m^6^A translational regulation of the SETD1A/B complex, which promotes the transcriptional activation mark, H3K4me3, and recruitment of the KLF1 transcription factor to erythroid gene promoters. Furthermore, these findings have potentially important implications for understanding myelodysplastic syndromes (MDS) as well as certain anemias.

## Results

### Human erythroleukemia (HEL) cells as a surrogate model of erythropoiesis

Since technical limitations precluded the use of human HSPCs for large scale functional genomic screens, we took advantage of HEL cells as a surrogate model, based on their expression of key markers and transcription factors associated with erythropoiesis^28^. We chose CD235a (GYPA) as the screen read-out, since this cell surface marker is the major sialoglycoprotein found on the erythrocyte membrane and is a faithful indicator of erythroid lineage maturation^29^. We first performed pilot studies with sgRNAs targeting *GYPA* and two transcriptional regulators of *GYPA* expression, GATA1 and LMO2^2,3,30^. Transduction of HEL cells with lentiviral (lv) vectors expressing sgRNAs targeting *GYPA*, *GATA1*, or *LMO2* resulted in significant reduction of CD235a expression (Supplementary Fig. 1a) and, for *sgGATA1* and *sgLMO2*, significant changes in gene expression of erythroid gene targets as well as erythroid progenitor stage-specific gene expression programs (Supplementary Fig. 1b, c). The results indicated that HEL cells can recapitulate at least a portion of the molecular features associated with erythropoiesis. In contrast to HEL cells, other leukemia cell lines with erythroid potential including K562 and TF-1 cells proved unsuitable for screening due to variable induction of erythroid cell surface markers.

### A genetic screen to identify regulators of erythropoiesis

**Fig. 1:**
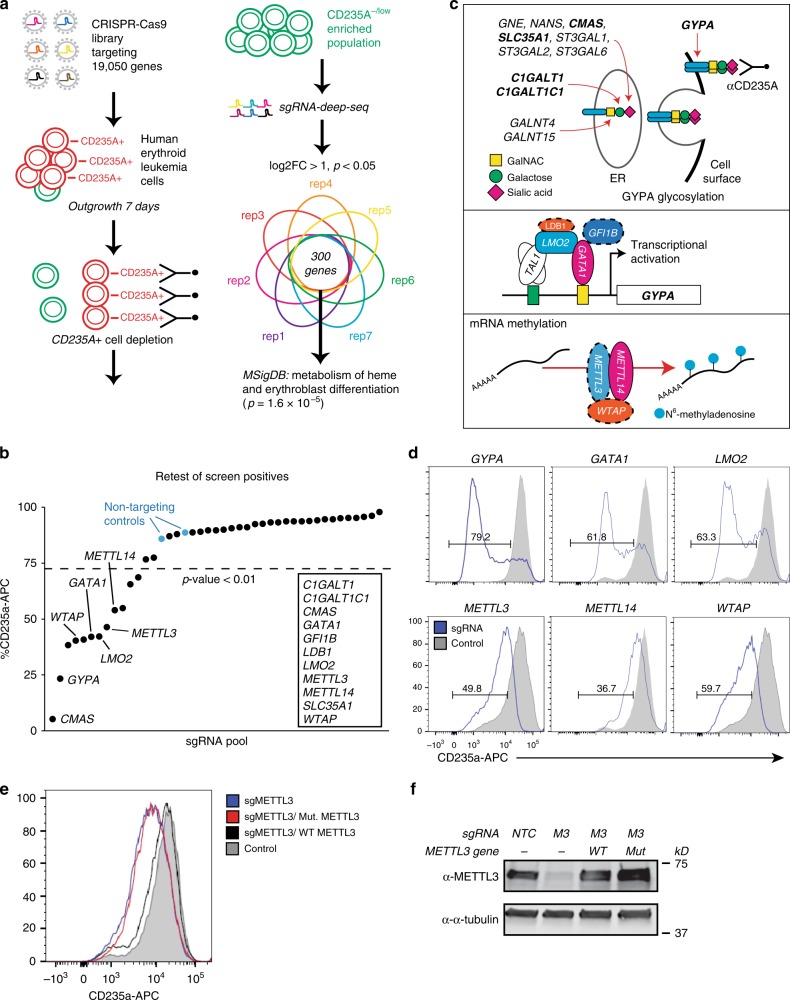
CRISPR–Cas9 whole-genome screening in HEL cells to identify regulators of erythropoiesis. **a** CRISPR–Cas9 screen design for enrichment of sgRNAs promoting the CD235a-/low state. **b** Individual genes retested by flow cytometry for CD235a surface expression on day 10 post-transduction in HEL cells. Cells were transduced with individual gene retests pools of four sgRNAs (see Methods for details). A modified *z*-score cutoff for a *p*-value < 0.01 was used to define a positive hit, with all scoring genes indicated within the box. For the m^6^A MTase complex, only *METTL14* scored in the primary screen. We found that the sgRNAs targeting *METTL3* and *WTAP* in the screening library were not effective and substituted sgRNAs from the human CRISPR Brunello lentiviral pooled library for *METTL3* and *WTAP*. *LDB1* and *GFI1B* each scored with 1 sgRNA in the initial screen and new sgRNAs were generated, also from the Brunello library. **c** Diagram of the three primary categories of screen hits. Top panel shows genes with multiple sgRNA hits in bold; all others have a single sgRNA scoring. Middle and bottom panels: genes validated by secondary individual gene tests highlighted in color; solid lines indicate 2 or more sgRNAs scored from primary screen, while dashed lines indicate 0 or 1 sgRNAs scored from primary screen. **d** Representative flow cytometry results for positive retest hits in HEL cells. Cells were transduced with lentiCRISPRv2-mCherry virus and assayed by FACS 7–9 days later. (*n* = 3–5 biological replicates). **e** Representative flow cytometry results for METTL3 rescue experiments utilizing full-length WT METTL3 or a catalytically inactive mutant METTL3, in *sgMETTL3*-KO HEL cells. (*n* = 2 biological replicates, mean ± SEM. Student’s *t*-test two-sided, %CD235a: NTC 89 ± 2.2; sgMETTL3 71.4 ± 1.9, *p* = 0.026; sgMETTL3/WT METTL3 88.7 ± 1.8; sgMETTL3/Mut METTL3 67.1 ± 1.5, *p* = 0.01). **f** Western blot for *METTL3* sgRNA KO and expression levels following rescue with WT METTL3 or the catalytically inactive mutant

Next, we performed a pooled lv-based CRISPR–Cas9 screen targeting 19,050 genes and 1864 miRNAs^27^ in HEL cells using two rounds of antibody-based CD235a+ cell depletion to derive a population of CD235a−/low cells (day 12 post-infection) (Fig. 1a, and Supplementary Fig. 1d). CD235a−/low cells were then subjected to sgRNA-seq^31^ to identify sgRNAs enriched in this population compared to the starting population, as well as control cells which had been outgrown in culture for 12 days (Fig. 1a, and Supplementary Fig. 1e, Supplementary Data 1). Using the filter criteria shown in Supplementary Fig. 1f, we identified 31 candidate genes which were then individually retested with lv-sgRNAs for modulation of CD235a expression. A total of 12 genes retested in HEL cells (Fig. 1b), primarily falling into three categories: components of the glycosylation machinery^32^, a modification required for antibody recognition of CD235a^33^; components of the GATA1 transcriptional regulatory complex^2,3^; and, surprisingly, the m^6^A mRNA MTase^34,35^ complex, *METTL14*, *METTL3*, and *WTAP* (Fig. 1b–d). To further validate that loss of m^6^A marking was responsible for the GYPA phenotype, we performed rescue experiments in *METTL3*-KO cells. When expressed at close to endogenous levels, WT METTL3 fully rescued GYPA surface expression, while a catalytically inactive METTL3 mutant (395–398 DPPW to APPA)^15^ failed to rescue (Fig. 1e, f). Given this potential role for m^6^A-dependent regulation of erythropoiesis, we further examined the underlying biology and phenotypes associated with these latter hits.

### m^6^A-mRNAs of hematopoietic and erythroid regulators

**Fig. 2:**
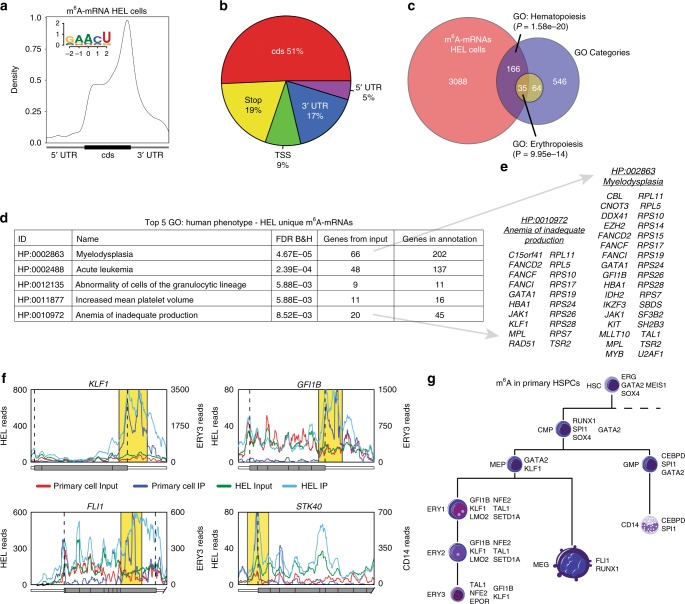
m^6^A marks the mRNA of key hematopoietic and erythroid regulators. **a** Distribution of m^6^A sites detected by meRIP-seq in HEL cells (150 µg), with an enrichment around the stop site, enriched m^6^A methylation site motif. **b** Pi chart displaying the frequency of m^6^A peaks, from HEL (150 µg), within different transcript regions: TSS, centered around translation start ATG; Stop, centered around the stop codon. **c** Genes uniquely methylated in HEL cells vs. 293T cells^36,37^ are enriched for key hematopoiesis and erythropoiesis genes. **d** Top gene ontology (GO) terms for HEL unique m^6^A mRNAs are enriched for genes with causal roles in hematopoietic diseases. **e** Gene callouts for GO terms identified in (**d**). **f** Transcript maps for erythropoiesis regulators detected as methylated in both HEL (150 µg) and adult BM cells show overlapping methylation patterns (yellow highlight). **g** Highlighting key hematopoietic regulators detected as methylated by meRIP-seq in flow sorted adult human BM cells. The parameters used to define the hematopoietic populations are outlined in Supplementary Fig. 2h

To reveal how m^6^A mRNA marks might affect erythroid regulatory networks, we performed methylated RNA immunoprecipitation sequencing (MeRIP-seq)^36,37^, which provides a site-specific read-out of m^6^A-modified transcripts. Profiling the polyA RNA m^6^A methylome of HEL cells revealed a total of 19,047 m^6^A peaks in 7266 protein-coding genes, representing 42.7% of genes expressed in HEL cells (Supplementary Data 2). The number of m^6^A peaks per gene ranged as high as 28, with 64.3% of m^6^A containing mRNAs having one or two peaks (Supplementary Fig. 2a). Consistent with previous MeRIP-seq results^36,37^, we observed enrichment of peaks around the stop codon of protein-coding mRNAs and a similar adenosine methylation site motif of GAACU, compared to the previously identified RRACH^37^ (Fig. 2a, b). Critically, m^6^A-marked mRNAs in HEL cells were enriched for regulators of hematopoiesis and erythropoiesis (e.g., *GATA1*, *FLI1*, *KLF1*, and *MPL*) (Fig. 2c) and genes with causal roles in erythroid-related diseases (Fig. 2d, e). The same m^6^A-marked mRNAs were not observed in previously published data from human embryonic kidney cells^37^ (Supplementary Fig. 2b, c).

To ensure relevance to normal cells, we also analyzed m^6^A-marked mRNAs in 9 flow sorted hematopoietic stem and progenitor cell populations (HSPCs) from freshly harvested adult human bone marrow. However, given the limited quantities of mRNA which can be isolated from these populations, an alternative to standard MeRIP-seq was required. We took advantage of an optimized protocol developed by He and colleagues for performing MeRIP-seq from as little as 2 µg of total RNA^38^, as opposed to the large total RNA quantities used for MeRIP-seq from our HEL cells (e.g., 100–300 µg)^36,37^. To validate the He protocol, we initially characterized the quality of the data by comparing the HEL cell results to the established MeRIP-seq protocol. Profiling the ribosomal RNA-depleted m^6^A methylome from 3 µg of HEL cell total RNA, we identified a total of 7282 m^6^A peaks in 3210 protein-coding genes (Supplementary Data 2). The distribution of the number of m^6^A peaks per gene was similar between the protocols with 67.4% vs. 72.7% of m^6^A containing mRNAs having one or two peaks (Supplementary Fig. 2a, d). We also observed a similar distribution of peaks within transcripts of protein-coding mRNAs and a similar adenosine methylation site motif of GGACU (Fig. 2a, b vs. Supplementary Fig. 2e, f). Overall, we observed a high level of agreement between the protocols, with a 94% overlap in methylated genes and a 76% overlap in called peaks identified with the He protocol (Supplementary Fig. 2g). However, this protocol under samples the m^6^A methylome (Supplementary Fig. 2g).

Applying this technique to 9 flow-sorted HSPC populations from freshly harvested adult human bone marrow (HSC, CMP, GMP, MEP, CD14, MEG, ERY1-3, Supplementary Fig. 2h lists the criteria used to define each population), we observe that 80.0% of the detected m^6^A methylated mRNAs in the HSPC populations overlapped with the HEL cells, with individual populations ranging from 92.3% overlap in the ERY1 population to 63.9% in the ERY3 population (Supplementary Fig. 2h). The specific m^6^A peaks detected in the HSPCs also had a high degree of overlap with those observed in the HEL cells (Fig. 2f). Consistent with the HEL cells, the m^6^A-marked mRNAs in the HSPCs were enriched for regulators of hematopoiesis and erythropoiesis (e.g., *GATA2*, *FLI1*, *KLF1*, and *SPI1*), reinforcing the notion that m^6^A likely has a role in regulating a variety of stages of hematopoiesis, including erythropoiesis (Fig. 2g).

### m^6^A-dependent regulation of erythroid gene expression

**Fig. 3:**
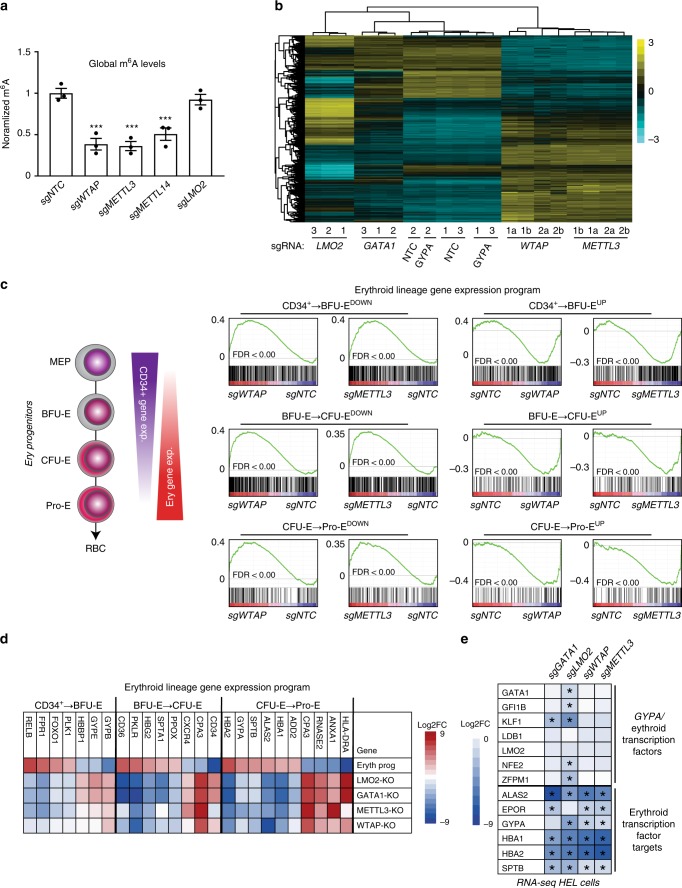
m^6^A-dependent regulation of erythroid gene expression programs. **a** Global m^6^A levels quantified by colorimetric assay in lv-sgRNA-KO transduced HEL cells are significantly reduced following *WTAP*-KO, *METTL3*-KO and *METTL14*-KO, and unchanged following *LMO2*-KO (*n* = 3, mean ± SEM. Student’s *t*-test two-sided, *** indicates *p* < 0.001). **b** A heatmap for RNA-seq analysis of HEL cells following KO of *METTLE3* and *WTAP*, as well as select erythroid genes, shows clustering of the two components of the m^6^A MTase complex and a unique transcriptional profile vs. *GATA1* and *LMO2*-KO. **c** GSEA analysis of *METTL3*-KO and *WTAP*-KO HEL cell RNA-seq, using custom gene sets for transcriptionally up and down genes during erythropoiesis as defined by ref. ^40^, shows enrichment for genes upregulated following m^6^A loss in HEL cells, which are downregulated during erythropoiesis and genes that are downregulated following m^6^A loss in HEL cells, which are upregulated during erythropoiesis. **d** A heatmap of transcriptional changes observed during normal erythropoiesis compared to m^6^A MTase KO HEL cells and KO HEL cells for select erythroid transcriptional regulators shows similar inverse patterns of gene expression. **e** A heatmap of gene expression changes from RNA-seq data for select erythroid transcriptional regulators and their targets from *sgGATA1*-KO, *sgLMO2*-KO, *sgWTAP*-KO, and *sgMETTL3*-KO HEL cells, showing decreased expression of the target genes in the absence of changes in transcription factor expression following m^6^A loss. The complete dataset is available in Supplementary Data 3. *Indicates significant changes relative to non-targeting control (sgNTC) (FDR < 0.05)

We next examined changes to steady-state mRNAs levels in HEL cells following m^6^A MTase inhibition. We first assayed m^6^A RNA levels after sgRNA-dependent targeting of *WTAP*, *METTL3*, *METTL14*, and *LMO2*. Inhibiting each of the m^6^A MTase subunits, but not *LMO2*, resulted in similar marked loss of m^6^A RNA levels in HEL cells (Fig. 3a). Consistent with this result, KO of *WTAP* and *METTL3* resulted in highly similar changes in steady-state mRNA levels in HEL cells (*R* = 0.837) (Fig. 3b; Supplementary Data 3). We found 3223 mRNAs significantly changed after *METTL3*-KO via RNA-seq (FDR < 0.01), with 1947 up and 1276 downregulated, and 2800 mRNAs significantly changed after *WTAP* KO via RNA-seq (FDR < 0.01), with 1774 up and 1026 downregulated (Supplementary Data 3). Importantly, these changes were not due use of sgRNA:Cas9, as sgGYPA only resulted in four significantly changed genes, three of which were the glycophorin paralogs GYPA, GYPB, and GYPE, with a common sgRNA target site (Supplementary Data 3).

GO analysis of the overlap between downregulated transcripts in *METTL3* and *WTAP* KO cells revealed modest enrichment outside of erythroid-related categories, including 40 genes involved in purine ribonucleoside triphosphate metabolic process, and those involved in mitochondrial electron transport and ATP production in mitochondria (Supplementary Fig. 3b, Supplementary Data 3). Analysis of upregulated transcripts detected enrichment for cytokine signaling, MHC class II/ immune genes, and transcription factor activity (Supplementary Fig. 3c). The latter category includes HSPC transcriptional regulators such as BCL11A, GFI1, and MYB, which has been shown to regulate expression of fetal hemoglobin^39^. More broadly, the upregulated genes represent a myeloid/myeloid progenitor expression profile (Supplementary Fig. 3c).

We next examined how *WTAP* and *METTL3*-KO affected erythroid progenitor stage-specific gene expression programs (Fig. 3c). Remarkably, we observed a highly similar and significant pattern of altered regulation of genes which are up or downregulated during the BFU-E, CFU-E, and Pro-E stages of erythropoiesis^40^ following *METTL3 and WTAP* KO (Fig. 3c, d), similar to *LMO2*-KO (Fig. 3d, Supplementary Fig. 1b, c, Supplementary Data 7). Genes that are normal increased in expression are suppressed and vice-versa.

We wondered whether the group of m^6^A marked and downregulated genes would contain key erythroid transcription factors, such as *GATA1*, *GFI1B*, *KLF1*, *LDB1*, *LMO2*, *NFE2*, and *ZFPM1*. While expression of many of these transcription factors were lower in *GATA1* or *LMO2*-KO cells, their expression did not significantly change in *WTAP* or *METTL3*-KO cells, despite the levels of many of their transcriptional targets dropping (Fig. 3e).

Further, we did not observe general enrichment for m^6^A marks in either up or down mRNAs, for example, downregulated mRNAs had a similar proportion of m^6^A marking (39.6% overall vs. 38% for *WTAP or* 40.2% for *METTL3*-KO). This suggested that changes in steady-state mRNAs in *WTAP* and *METTL3*-KOs were not broadly due to changes in mRNA turnover. We further examined mRNA turnover of five genes with m^6^A mRNA marks including two transcription factors, *GATA1* and *FLI1*, and three genes that we characterize below, including *CXXC1*, *PABPC1*, and *PABPC4* (Supplementary Fig. 3d). KO of *METTL3*, *METTL14*, or *WTAP* did not significantly affect their half-life in HEL cells.

Taken together, these results suggested that m^6^A MTase activity may affect expression of erythroid genes independently of affecting steady-state mRNA levels of erythroid transcriptional regulators and that m^6^A MTase activity suppresses myeloid lineage genes in HEL cells.

We next asked whether *METTL3 and WTAP* KO-driven changes in erythroid gene expression could be due to altered splicing. It was previously observed that m^6^A marks may promote exon inclusion in certain mRNAs^17,20^. To this end, we examined changes in all predicted exon–exon and exon–intron boundaries (using MISO^41^ and MATS^42^) and compared any altered splicing patterns to m^6^A-marked transcripts. While both analysis tools revealed significant splicing changes in hundreds of genes after *METTL3 and WTAP* KO, we did not observe significant overlap between m^6^A peaks at exon/intron boundaries, either in general or for specific classes of splicing events (Supplementary Fig. 3e) (Supplementary Data 4). This suggested that m^6^A-induced changes in splicing were not driving our phenotypes.

### m^6^A-dependent translation regulation of erythroid genes

**Fig. 4:**
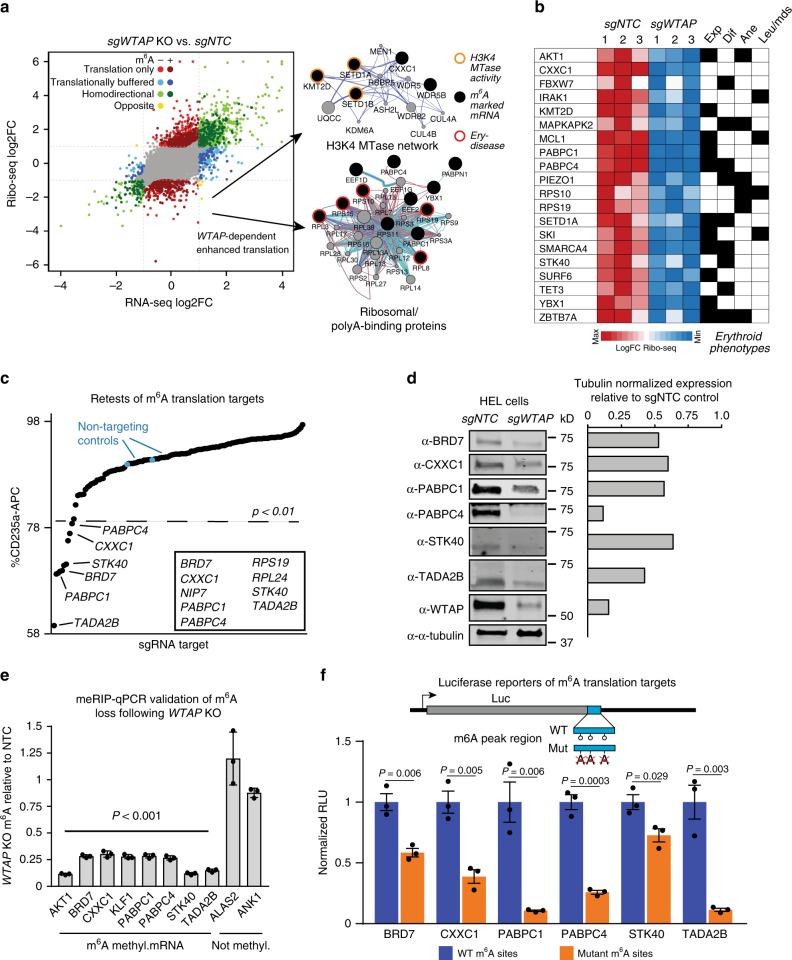
m^6^A-dependent translation regulation of known or putative erythropoiesis regulators. **a** Translational and transcriptional changes in *WTAP*-KO HEL cells measured by ribosome profiling, network maps for interactions enriched in m^6^A uniquely translationally down genes highlight targeting of the H3K4 MTase complex, as well as ribosomal proteins. **b** A heatmap of genes uniquely translationally down with known or suspected roles in hematopoietic progenitor cell function (exp), erythropoiesis (dif), anemia (ane), and/or other hematopoietic diseases (leu/mds). These and other genes in this category, along with associated references are in Supplementary Data 5. **c** Several m^6^A targets, which are only translationally changed following *WTAP*-KO, can partially recapitulate the m^6^A-MTase inhibition phenotype in HEL cells when individually targeted by sgRNAs or shRNAs. Several genes that scored as essential in the HEL cell outgrowth CRISPR–Cas9 screen (Supplementary Data 1) were targeted with shRNAs rather than sgRNAs, including: *RPL3*, *RPL24*, *RPS19*, *RRS1*, *RUVBL2*, and *SFPQ*. Cells were infected with pools of four lv-sgRNA or individual shRNAs. A *z*-score cutoff for a *p*-value < 0.01 was used to define a positive hit, with all scoring genes indicated within the box. **d** Western blot validation of non-essential retest hits following *WTAP* KO in HEL cells shows reduced protein expression for all hits. **e** meRIP-qPCR validation of altered m^6^A levels for genes translationally decreased following *WTAP* KO relative to NTC m^6^A levels. (*n* = 3 technical replicates, mean ± SEM. *t*-test two-sided) **f** Luciferase reporter validation of translationally downregulated genes containing an m^6^A site following *WTAP* KO. The region identified by meRIP-seq as containing an m^6^A site and with the largest number of sites matching the RRACH motif wase fused to the 3′ end of luciferase, except for *STK40* with a 5′ fusion, and the central A of the motif mutated. The selected region for all targets showed m^6^A-dependent regulation of expression. (*n* = 3 biological replicates, mean ± SEM. *t*-test two-sided)

We next asked if m^6^A marking might impact the translation of genes responsible for promoting erythroid gene expression. For these experiments, we used *WTAP* KO, which phenocopies *METTL3*-KO in all practically measurable ways (e.g., gene expression changes, flow cytometry phenotypes, and m^6^A loss) (Figs. 1d, 3a–e). We utilized ribosome profiling^43,44^, to analyze *WTAP*-dependent effects on mRNA translation. This analysis yielded 1055 genes that were translationally changed without significant alterations to mRNA levels following *WTAP* KO, with 738 up and 317 downregulated (Fig. 4a and Supplementary Data 5). Of these, ~45% of up and ~63% of downregulated mRNAs were m^6^A methylated. Interestingly, more heavily m^6^A-marked mRNAs show significantly lower mRNA ribosome association after *WTAP* KO (Supplementary Fig. 4a), indicating that m^6^A marks are directly promoting translation of a subset of mRNAs. Supporting this notion, we did not observe global changes in de novo protein synthesis in *WTAP* KO cells, as determined by puromycin incorporation in nascent peptide chains (Supplementary Fig. 4b). Intriguingly, among translationally downregulated m^6^A-mRNAs after *WTAP* KO, we observed significant enrichment for proteins with RNA binding and histone methyltransferase activity (Supplementary Data 5), including ribosomal proteins with causal roles in human Diamond–Blackfan anemia^5,45^, and key H3K4 MTases and associated proteins, including catalytic MTases KMT2D/MLL4 (ref. ^46^), SETD1A^47^, and SETD1B^48^ (Fig. 4a). Furthermore, at least 48 m^6^A-regulated mRNAs in the translationally down category have known or suspected roles in erythropoiesis, erythroid-related diseases, and/or hematopoietic progenitor cell function, for example, MCL1 (ref. ^49^), FBXW7 (refs. ^50,51^), and KMT2D^52,53^ (Fig. 4b and Supplementary Data 5).

As the results suggested that m^6^A-dependent translation regulation of these mRNAs could explain our erythropoiesis phenotypes, we performed functional retests on 54 genes in the translationally down category for effects on CD235a expression in HEL cells (Fig. 4c). We found that inhibition of 9 of these genes significantly decreased CD235a expression (Fig. 4c): *BRD7*, *CXXC1*, *NIP7*, *PABPC1*, *PABPC4*, *RPS19*, *RPL24*, *STK40*, and *TADA2B*. Except for *BRD7* and *TADA2B*, each of these genes has known erythroid-related functions (Supplementary Data 5). For example, Pabpc4 has been shown to bind to mRNAs associated with erythroid differentiation in mouse leukemia cells and is required for maintaining the steady-state mRNA levels of a subset of these, including *CD235a/GYPA* mRNA^54^, while *Stk40* deletion leads to anemia in mouse embryos characterized by a reduction in progenitors capable of erythroid differentiation^55^. Further validation of BRD7, CXXC1, PABPC1, PABPC4, STK40, and TADA2B by Western blot showed that their steady-state protein levels decreased after *WTAP* KO (Fig. 4d). Significant reductions in m^6^A levels for these genes following *WTAP* KO compared to non-m^6^A containing mRNAs were also detected by MeRIP-qPCR (Fig. 4e).

To demonstrate a direct link between loss of m^6^A marking and translational down regulation, we generated luciferase reporters for BRD7, STK40, PABPC1, PABPC4, CXXC1, and TADA2B containing 197–438 bp of the m^6^A marked region with the largest number of m^6^A motifs. When tested in HEL cells, mutation of the putatively methylated A sites resulted in reduced reporter activity for all constructs (Fig. 4f).

These results demonstrate that m^6^A marking of discrete mRNA domains promotes the translation of genes with causal roles in promoting CD235a/GYPA expression and known or likely roles in erythropoiesis. The results further suggest that m^6^A mRNA regulatory elements are transferable to other mRNAs and do not depend on gene context (e.g., cis-acting chromatin binding factors specific to marked genes).

### m^6^A MTase activity regulates H3K4me3 promoter marking

**Fig. 5:**
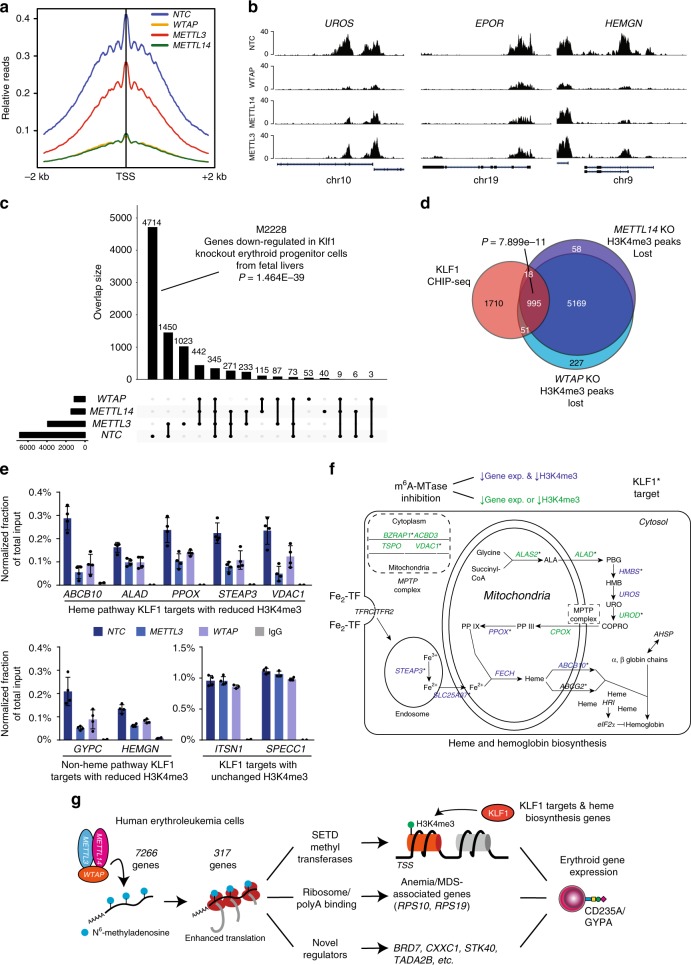
Promoter histone H3K4me3 marks are lost in the absence of m^6^A methyltransferase activity. **a** An H3K4me3 relative read distribution plot around the transcription start site (TSS) of methylated genes in CRISPR NTC, *WTAP*-KO, *METTL3*-KO, and *METTL14*-KO HEL cells. The plot shows a marked reduction in H3K4me3 marks following loss of *WTAP* and *METTL14* with a more modest effect with loss of *METTL3*. **b** Bedgraphs of normalized H3K4me3 CUT&RUN data for select genes with reduced methylation following m^6^A loss. **c** An upset plot of the H3K4me3 peaks found in CRISPR NTC, *WTAP*-KO, *METTL3*-KO, and *METTL14*-KO HEL cells. The pattern of H3K4me3 loss is consistent with loss of KLF1 transcriptional regulation. Genes with reduced H3K4me3 following loss of any of the three m^6^A MTase components are enriched for genes downregulated following *Klf1* KO. **d** A Venn diagram showing enrichment for KLF1 CHIP-seq target genes^63^ among the H3K4me3 peaks lost following *WTAP*-KO or *METTL14*-KO, suggesting m^6^A-mediated epigenetic regulation of the KLF1 transcriptional program. **e** KLF1 CHIP-qPCR validation of several genes with or without altered H3K4me3 levels following *METTL3*-KO and *WTAP*-KO, (*n* = 4, mean ± SEM). **f** A diagram of the iron procurement, heme synthesis and transport, and hemoglobin assembly in erythroid cells, highlighting regulation by KLF1 and altered H3K4me3 marking following m^6^A loss. *: KLF1 CHIP-seq target genes^63^; Blue: reduced H3K4me3 marking and transcript expression following m^6^A-MTase inhibition; Green: reduced H3K4me3 marking or transcript expression following m^6^A-MTase inhibition. These results highlight inhibition of multiple pathways involved in hemoglobin synthesis following loss of m^6^A possibly through down regulation of the KLF1 transcriptional program. **g** The proposed model for the role of m^6^A in translational regulation of erythroid gene expression and erythropoiesis

Because we observed that WTAP activity promoted translation of a histone H3K4 MTase network, e.g., *SETD1A*, *SETD1B*, and *KMT2D* (Fig. 4a), we wondered whether H3K4 methylation would be dependent on m^6^A methyltransferase activity. Previous work has shown that mice deficient for *Setd1a* show loss of promoter-associated H3K4 methylation in the erythroid lineage, loss of erythroid gene transcription, and blockade of erythroid differentiation^56^. Further, KMT2D has been shown to localize to and regulate transcription of the β-globin locus^52^. Because SETD1A and SETD1B have established roles in maintaining and promoting H3K4me3 and this mark is strongly associated with transcriptionally active promoters^57,58^, we focused on analysis of H3K4me3 after loss of m^6^A MTase activity. To this end, we performed CUT&RUN^59^ to map H3K4me3 marks in *METTL14, METTL3*, and *WTAP* KO HEL cells. By this technique, we observe 6871 H3K4me3 marked genes centered around transcription start sites (TSS) in HEL cells. Critically, we observed marked loss of this signal in *METTL14, METTL3*, and *WTAP* KO cells, with a much larger loss observed with *METTL14* and *WTAP* KOs, 1461 and 1127 H3K4me3 marked genes, respectively (Fig. 5a, c). Examples of some promoters with loss of H3K4me3 are shown in Fig. 5b, including *UROS*, a key constituent of the heme biosynthetic pathway^60^, *EPOR*, the receptor for erythropoietin, a growth factor critical for normal erythropoiesis, and *HEMGN*, a GATA1 transcriptional target^61^.

A more general examination of H3K4me3 loci lost after m^6^A-MTase deletion, revealed marked enrichment for genes that are downregulated in mouse erythroid progenitors that have deletion of *Klf1* (Fig. 5c) (GO ID M2228; *p*-value = 1.464E−39)^62^. KLF1 is a master transcriptional regulator of erythropoiesis, facilitating many aspects of terminal erythroid differentiation including production of alpha- and beta-globin, heme biosynthesis, and coordination of proliferation and anti-apoptotic pathways^63^. However, as mentioned above, we did not observe down regulation of *KLF1* in HEL cells after *METTL14, METTL3*, or *WTAP* KO. So, we compared the promoters that had lost H3K4me3 after KO of m^6^A-MTase activity with those previously shown to be directly bound by KLF1 in primary erythroid progenitors^63^. Remarkably, 36% of KLF1 bound promoters lost H3K4me3 marking after inhibition of *METTL14* and *WTAP* (*p*-value = 7.899e−11) (Fig. 5d). Further validating this observation, we found that KLF1 binding was significantly reduced at target genes with low H3K4me3 after m^6^A inhibition with an average reduction in KLF1 binding of 67.7% (53.6–78.9%) for *METTL3*-KO and 57.5% (39.2–76.9%) for *WTAP* KO relative to NTC control cells, as measured by ChIP-qPCR (Fig. 5e). No change in binding was observed at target sites with unchanged H3K4me3 levels 84.9–109.2% (Fig. 5e).

One of the key roles of KLF1 in erythropoiesis is activating expression of genes directly involved in heme and hemoglobin biosynthesis^63,64^. We observe that m^6^A-MTase activity is required for both maintenance of promoter H3K4me3 and expression of most of the genes in this pathway (Fig. 5f), further suggestive of a critical link between m^6^A regulation and RBC function.

In addition to KLF1 targets, we also examined H3K4me3 status of genes expressed during CD34 to BFU and CFU to Pro-E stages of erythropoiesis from our previous analysis (Fig. 3). We also found that a significant number of these genes lose H3K4me3 peaks after m^6^A-MTase inhibition (Supplementary Data 6), again underscoring the importance of m^6^A-mRNA regulation in promoting epigenetic marking of erythroid genes. A gene ontology analysis of the non-KLF1 target genes with reduced H3K4me3 marks revealed that the majority fall into three main categories: (1) RNA binding and processing; (2) translation and ribosome biogenesis; (3) cell cycle (Supplementary Data 6). In addition, there is enrichment for genes potentially regulated by the transcription factor ELK1 (Supplementary Data 6).

Combined, these data suggest a model where by m^6^A marks promote the translation of a broad network of genes required for erythrocyte specification and maturation (Fig. 5g). The target genes can largely be split into three distinct groups which when perturbed may lead to disrupted erythropoiesis: (1) H3K4me3 regulation of the KLF1 transcriptional program required for development of early erythroid progenitors and regulation of heme synthesis and hemoglobin assembly; (2) ribosomal proteins and regulators of mRNA stability; and (3) genes with undescribed functions in erythropoiesis.

### m^6^A loss blocks erythropoiesis in human bone marrow HSPCs

**Fig. 6:**
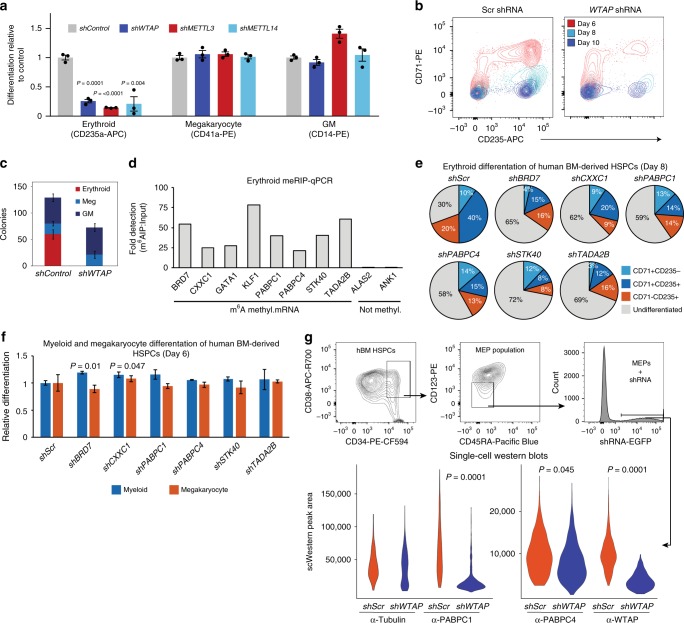
Knockdown of the m^6^A MTase complex blocks erythropoiesis in human adult, bone marrow-derived HSPCs. **a** Flow cytometry of *WTAP* KD, *METTL3*-KD, and *METTL14*-KD hBM HSPCs differentiated in liquid culture reveals a block to erythropoiesis with no impact on megakaryopoiesis and an enhancement of myelopoiesis with *METTL3*-KD (*n* = 3, mean ± SEM, *t*-test two-sided). **b** Flow cytometry of erythroid progenitor populations reveals an early block to erythropoiesis following *WTAP* KD. At day 6, *WTAP* KD CD235+/CD71+ cell numbers were only 8.4% of scr control and by day 10, *WTAP* KD CD235+/CD71− cell numbers decreased further to 5.5% of control cells. **c** Colony formation assays from *WTAP* KD hBM HSPCs confirm the block to erythropoiesis (*n* = 6, mean ± s.d., erythroid *t*-test two-sided, *p* < 0.0000001). **d** MeRIP-qPCR validation of m^6^A methylation for key erythroid regulators and genes translationally decreased following *WTAP* KO in a bulk population of adult BM erythroid cells (ERY1, ERY2, and ERY3) pooled from six donors. (*n* = 1). **e** Flow cytometry of lv-shRNA-KD of the translationally altered genes identified in Fig. 4c in hBM HSPCs differentiated in erythroid promoting liquid culture reveals a delay or reduction in erythropoiesis at day 8. (*n* = 2, mean). **f** Flow cytometry for differentiation potential of hBM CD34 + HSPCs transduced with lv-shRNA of the translationally altered genes identified in Fig. 4c and cultured in megakaryocyte or myeloid liquid culture differentiation conditions shows no effect on those lineages, except for enhanced myelopoiesis following *CXXC1* KD and *BRD7* KD (*n* = 2, mean ± range, *t*-test two-sided). **g** Single-cell western blot analysis of *WTAP* KD MEPs recapitulates the reduction in PABPC1 and PABPC4 protein observed in *WTAP* KO HEL cells (data from a minimum of 250 cells were used per sample, Kolmogorov–Smirnov)

To demonstrate that m^6^A MTase function impacts erythroid lineage specification, we knocked-down (KD) *METTL14, METTL3*, and *WTAP* in adult human bone marrow-derived CD34+ HSPCs with lv-shRNAs and assessed lineage formation in vitro (Fig. 6 and Supplementary Fig. 5a–g). In suspension cultures promoting erythroid lineage formation, we observed a near total loss of CD235a+ cells in m^6^A-MTase KD cells (Fig. 6a and Supplementary Fig. 5a–c). There was no observable impact on megakaryocytic differentiation (Fig. 6a and Supplementary Fig. 5a–c). For myeloid differentiation, there was no observable change for *WTAP*-KD or *METTL14*-KD and an increase, though not statistically significant, from 37.5 ± 1.8 in Scr control to 52.3 ± 5.0 (*p*-value = 0.1062) with *METTL3*-KD (Fig. 6a and Supplementary Fig. 5a–c). Further, examination of the sequential appearance of CD71+ and CD235a+ cells during early-mid and mid-late erythroid differentiation^65^ revealed that few erythroid progenitors progressed past the earliest stages of erythropoiesis following *WTAP, METTL3,* or *METTL14*-KD (Fig. 6b, Supplementary Fig. 5a, b). Consistent with these findings, *WTAP*-KD resulted in a complete loss of erythropoiesis in colony formation assays, including burst and colony forming units (i.e., BFU-E and CFU-E)^66^, without a noticeable effect on megakaryocyte or myeloid colony formation (Fig. 6c). Within the MEP population (CD34+CD38+CD45RA−CD123−)^67^, *WTAP* KD did not alter the number of CD41− erythroid or CD41+ megakaryocyte committed progenitors (Supplementary Fig. 5h). Thus, the results are consistent with m^6^A-MTase activity being required for early erythropoiesis in adult hematopoietic progenitors just after the MEP stage.

### Regulation of m^6^A mRNA targets in bone marrow HSPCs

We next wished to determine whether key targets of m^6^A mRNA translation regulation in HEL cells, including *BRD7*, *CXXC1*, *PABPC1*, *PABPC4*, *STK40*, and *TADA2B*, show similar regulation in human erythroid progenitor populations and have causal roles in promoting CD235a/GYPA expression and erythroid lineage formation. To this end, we first examined their m^6^A-mRNA status using RNA isolated from bulk erythroid progenitor populations, consisting of CD34−CD71+CD235a−, CD34-CD71+CD235a+ and CD34−CD71−CD235a+ cells and performed MeRIP-qPCR. We included genes which are not m^6^A marked in HEL cells, *ALAS2* and *ANK1*, as well as erythroid transcription factors which are m^6^A marked in HEL cells, *GATA1* and *KLF1* (Fig. 6d). By this approach, we detected significant m^6^A signal from *BRD7*, *CXXC1*, *PABPC1*, *PABPC4*, *STK40*, and *TADA2B* (21.2-fold to 60.9-fold enrichment vs. total RNA) and *GATA1* and *KLF1* (27.7-fold and 78.4-fold enrichment, respectively), with minimal background detection of the unmethylated targets (0.01-fold to 0.10-fold detection vs. total RNA) (Fig. 6d).

Next, we utilized lv-shRNAs to KD *BRD7*, *CXXC1*, *PABPC1*, *PABPC4*, *STK40*, and *TADA2B* in CD34+ adult BM cells and then assessed their impact on hematopoietic differentiation in liquid culture. KD of each of the genes strongly delayed the emergence of erythroid progenitors (~3 days), without negatively affecting megakaryocyte or monocyte lineage formation (Fig. 6e, f). Furthermore, none of the KDs produced significant differences in megakaryocytic differentiation, and only KD of *CXXC1* and *BRD7* produced a small but significant increase in myeloid differentiation (Fig. 6f). These results demonstrate that m^6^A-MTase mRNA targets are also found to be m^6^A-regulated in primary erythroid progenitors and that their partial inhibition blocks or delays human erythroid lineage specification.

### *WTAP* is required for PABPC1 and PABPC4 expression in MEPs

We next tested whether m^6^A-mRNA regulation may affect translation regulation of *BRD7*, *CXXC1*, *PABPC1*, *PABPC4*, *STK40*, and *TADA2B* in human HSPCs. However, because m^6^A-MTase inhibition blocks the appearance of erythroid progenitors in human HSPCs, and due to the paucity of cells in progenitor populations from human donors, traditional western-blotting is not possible. Instead, we used single-cell Western blotting to examine the effects of *WTAP* KD on protein expression in flow sorted MEP populations, where only hundreds of single cells are needed (Supplementary Fig. 6b, c). Unfortunately, only PABPC1 and PABPC4 proteins were detectable at measurable levels using this assay (Fig. 6g and Supplementary Fig. 6c). Both *PABPC1* and *PABPC4* encode cytoplasmic polyA binding proteins shown to promote the expression of β-globin in erythroid-differentiated CD34 + cells^68^ and promote the erythroid maturation of mouse erythroid leukemia cells^54^, respectively. By this analysis, targeted lv-based KD of *WTAP* in MEP-enriched populations caused significant reduction in both PABPC1 and PABPC4 protein, but not control beta-tubulin. We conclude that WTAP activity promotes their protein expression in human HSPCs and likely does so through m^6^A-mRNA regulation (Fig. 6g).

## Discussion

Here, we report the discovery of a form of post-transcriptional regulation of human erythroid gene expression and lineage specification, m^6^A-mRNA marking. From whole-genome CRISPR–Cas9 screening, we found that the three core components of the m^6^A MTase complex (*METTL14*, *METTL3*, and *WTAP*) are required for maintaining CD235a/GYPA expression in HEL cells and erythroid lineage specification in human HSPCs. Follow-up studies revealed that m^6^A-MTase complex activity and m^6^A marking are required to promote the expression of genes associated with stage-specific erythroid progenitor transcription programs (Fig. 3c, d).

Mechanistically, we observed that m^6^A mRNA regulation promotes the translation of hundreds of genes, many of which have causal roles in erythropoiesis and erythroid-associated diseases or appeared in two prominent gene networks: histone H3K4 MTases and interacting proteins, and ribosome/RNA binding proteins (Fig. 4a).

Comprehensive validation of m^6^A mRNA targets translationally regulated in HEL cells reveals that *BRD7*, *CXXC1*, *NIP7*, *PABPC1*, *PABPC4*, *RPS19*, *RPL24*, *STK40*, and *TADA2B* are required for promoting CD235a/GYPA expression and are required for erythroid lineage specification in hHPSCs (Figs. 4, 6). Among these, the polyA RNA binding proteins *PABPC1* and *PABPC4* are of note, *PABPC1* has been shown to bind to and stablize the *β-Globin/HBB* mRNA by inhibiting its deadenylation in erythroid precursors^68^, whereas *PABPC4* has been implicated in binding to and stabilizing *GYPA* mRNA, as well as other erythroid targets, including *α-Globin/HBA1/HBA2*, *β-Globin/HBB*, *BTG2*, and *SLC4A1* (ref. ^54^). These activities could, in theory, oppose the activity of the m^6^A-mRNA binding protein YTHDF2, which can mediate mRNA decay^16^.

In addition, NIP7 has been previously implicated in 18S rRNA maturation and the MDS-associated Shwachman–Bodian–Diamond syndrome^69^. *RPS19* encodes a ribosomal protein that when heterozygous for a loss of function mutation causes DBA^70^. *STK40* codes for a serine threonine kinase required for definitive erythropoiesis just after the MEP state in the fetal mouse liver, similar to our m^6^A-MTase inhibition phenotype in hHSPCs^55^. The other genes have not been previously implicated in erythropoiesis, including: *BRD7*, a member of the bromodomain-containing protein family implicated in tumor suppression of p53 and PI3K pathways^71,72^; *CXXC1*, which encodes a key regulator of H3K4 histone methylation, cytosine methylation, cellular differentiation, and vertebrate development^73^; and *TADA2B*, which encodes a transcriptional adapter for transcriptional activation factors^74^.

Regarding m^6^A-mRNA-dependent regulation of SETD1A/B H3K4 MTase activity, we find that m^6^A-MTase activity is required for maintaining H3K4me3 marks at erythroid-related genes promoters. This included KLF1-dependent transcription targets and the heme and hemoglobin synthesis pathway (Fig. 5). SETD1A and KMT2D have previously been shown to have key roles in regulating erythroid gene transcription, and, at least for SETD1A, key roles in erythroid lineage specification^52,56^. Our results suggest that m^6^A marks have key roles in maintaining optimal H3K4me3 marking at erythroid gene targets and, as a result, proper expression of lineage genes.

Phenotypically, in adult human HPCs, our studies show that m^6^A MTase function is critical just after the MEP stage of erythroid lineage specification. Intriguingly, key genes associated with impaired erythropoiesis, including drivers of Diamond–Blackfan Anemia (DBA) and MDS^5^, are m^6^A targets and require m^6^A-MTAase activity for their protein expression. Loss of these genes activities may explain the maturation failure of the small numbers of detected early erythroid progenitors (Fig. 6). We detected m^6^A mRNA methylation in 70 out of 104 known/putative MDS genes, including 8 of the top 10 most frequently mutated genes (*RPS19*, *TET2*, *SF3B1*, *ASXL1*, *RUNX1*, *DNMT3A*, *ZRSR2*, and *STAG2*)^75^. Further, *RPS19*, the most commonly mutated gene in DBA (~25%) is decreased translationally following *WTAP* KO, as is the less frequently mutated *RPS10* (~2.6%)^70^. This suggests that m^6^A mRNA regulation could emerge as an erythroid-associated disease modifier.

Previous work has established three prominent types of post-transcriptional mRNA regulation impacting erythropoiesis or expression of key erythroid genes. These include: microRNA-based targeting of critical pro- or anti-erythroid transcription factors (rev. in ref. ^76^); erythroid-specific messenger ribonucleoprotein-dependent regulation of *β-Globin/HBB* mRNA stability^68^; and the iron regulatory protein/iron-responsive element regulatory system, which post-transcriptionally controls the translation of several key heme biosynthesis genes^77,78^. Our results provide insight into another critically, yet unexpected, layer of post-transcriptional mRNA regulation in erythroid cells with several points of intersection with these other forms of regulation.

However, there are several lingering questions from our results. One is what role m^6^A marking may have on important hematopoietic and erythroid transcription factors. We find evidence in both HEL cells and primary hematopoietic cell populations that, *GATA1*, *GATA2*, *KLF1*, *RUNX1*, and *SPI1* mRNAs are m^6^A methylated (Fig. 2). However, in HEL cells this regulation does not appear to affect steady-state mRNA levels or their translation, as read-out by RNA-seq and Ribo-seq, respectively. Future studies will need to address roles of m^6^A-dependent regulation of these factors in primary erythroid cells and whether oncogenic transformation affects this regulation.

A more general question raised by our data is how specificity of m^6^A-mRNA marking is achieved. In line with other m^6^A datasets, we observe that 42.7% of genes in HEL have m^6^A-marks associated with their mRNAs. Many of these genes display erythroid-specific gene expression, suggesting lineage-specific determinants of specificity. The source of this apparent specificity, whether through specific factors or other regulatory mechanism remains unknown.

Similarly, m^6^A-mediated translation regulation may be dependent on lineage-specific factors. The current model for m^6^A-mediated translational regulation by YTHDF1/YTHDF3 is dependent on METTL3 mediating the formation of an mRNA loop structure to facilitate YTHDF1 recruitment of eIF3 (ref. ^22^). This interaction, however, brings the distal regions of the 3′ UTR into proximity with eIF3, while the majority of 3′ UTR m^6^A sites are proximal. Therefore, additional factors are likely involved to facilitate YTHDF1/eIF3 interactions, some of which may prove to drive lineage-specific translational regulation.

Taken together, our results reveal a physiological role for m^6^A-dependent regulation of mRNA translation in maintaining erythroid gene expression programs and promoting erythroid lineage specification.

## Methods

### Cloning

sgRNAs were cloned into lentiCRISPRv2 puro vector (Addgene) and lentiCRISPRv2-mCherry (a variant created by excising puro with NheI and MluI, followed by addition of P2A-mCherry by Gibson Assembly). Individual gene pools of 4 sgRNAs were cloned by first PCR amplifying the individual sgRNAs from oligo template using Phusion High-Fidelity DNA Polymerase (NEB), followed by cleanup with the PureLink PCR Purification Kit (Invitrogen). The following primers were used for sgRNA amplification: Array_F-TAACTTGAAAGTATTTCGATTTCTTGGCTTTATATATCTTGTGGAAAGGACGAAACACCG and Array_R-ACTTTTTCAAGTTGATAACGGACTAGCCTTATTTTAACTTGCTATTTCTAGCTCTAAAAC. The oligo template is a 60-mer oligo: GTGGAAAGGACGAAACACCg- sgRNA sequence – GTTTTAGAGCTAGAAATAGC. The individual sgRNA PCR products were quantified on a Nanodrop 1000 (ThermoFisher) and pooled in equal amounts. The PCR product pools were then cloned into the Esp3I site of lentiCRISPRv2 by Gibson Assembly. Four colonies from each pool were sequenced to validate the pool identity, while the remainder of the plate was scraped, and DNA prepared using the NucleoBond Xtra kit (Macherey–Nagel).

For cloning shRNAs, a modified pLL3.7 vector (pLL3.7-EF1a-mini) was generated replacing the CMV promoter with EF1α and inserting an Esp3I cloning cassette. The CMV promoter was removed by *Nsi*I and *Age*I digest and the EF1α promoter inserted by Gibson Assembly. A new shRNA cloning site was added by digesting with *Xho*I and *Nsi*I and inserting a 100-bp cassette containing Esp3I sites on both ends by Gibson Assembly. All shRNAs were cloned into the pLL3.7-EF1a-mini vector, primarily following the Genetic Perturbation Platform shRNA/sgRNA Cloning Protocol, (portals.broadinstitute.org/gpp/public/resources/protocols) with one modification. Due to the altered cloning site, the format for the annealed oligos changed to the follow: 5′ GTTT--- 21 bp Sense --- CTCGAG --- 21 bp Antisense --- TTTTTG 3′ and 5′ GCCGCAAAAA --- 21 bp Sense --- CTCGAG --- 21 bp Antisense --- 3′. In brief, oligos are re-suspend in ddH20 and annealed in the following reaction: 1.5 μL of forward oligo (100uM); 1.5 μL of reverse oligo (100uM); 5 μL of 10× NEB buffer 2; 42 μL ddH_2_O.

The reaction is incubated for 4 min at 95 °C, and then 70 °C for 10 min, followed by a slow ramp to RT over a couple of hours. The vector is linearized with Esp3I and gel purified using the Monarch Gel Extraction Kit (NEB) or Zymoclean Gel DNA Recovery Kit (Zymo Research). 1 μL of annealed oligos were then ligated into the Esp3I site of 20 ng of pLL3.7-EF1a-mini with T4 ligase (NEB) for 4 h at 16 °C. All sgRNA and shRNA sequences can be found in Supplementary Data 8.

The luciferase reporters were generated by cloning regions of potential m^6^A regulation into the pPIG vector (this paper). The m^6^A regions of interest were ordered as either WT or mutant, with all central adenines within the m^6^A methylation motif mutated, gBlocks (IDT). Luciferase was PCR amplified out of Luciferase-pcDNA3. Luciferase and the reporter region were cloned between the *Not*I and *Mlu*I sites of pPIG by Gibson Assembly. pPIG is heavily modified version of pGIPZ with Hygro resistance removed and the region between the *Xba*I and *Mlu*I sites replaced with the following cassette, XbaI – hPGK promoter – NotI – IRES – AgeI – EGFP – MluI. The sequences for the cloned regions and mutated potential m^6^A sites can be found in Supplementary Data 8.

*METTL3* expression vectors were generated by PCR amplification of full-length wild-type *METTL3* out of HEL cell cDNA and sequence verified. The catalytically inactive *METTL3* mutant (395–398 DPPW to APPA)^15^ was synthesized as a gBlock (IDT). *METTL3* and *METTL3* mutant were cloned between the NotI and MluI sites of pPIG by Gibson Assembly.

### Cell culture and lentiviral transduction

HEL cells (Papayannopoulou lab, Univ. of Washington) were grown in RPMI-1640 (ThermoFisher) supplemented with 10% fetal bovine serum (FBS) at 37 °C and 5% CO_2_ between 400,000 and 2 million cells/mL. 293T (ATCC) cells were grown in Dulbecco’s modified Eagle’s medium (ThermoFisher) supplemented with 10% FBS at 37 °C and 5% CO_2_. For lentiviral transduction, HEL cells were plated at 500,000 cells/mL in RPMI-1640 + 10% FBS, 8 μg/mL protamine sulfate (Fisher Scientific ICN19472905) and virus was added at the MOIs described below. After 48 h, the cells are washed with 1× PBS and fresh RPMI + 10% FBS added.

### Virus production

The lentiCRISPRv2 vector was used for all CRISPR–Cas9 experiments, the pLL3.7-EF1α vector was used for all RNAi experiments and pPIG for all other experiments. For virus production, 12 µg of either lentiCRISPRv2 or pLL3.7-EF1a, 8 µg of psPAX and 3 µg of pMD2.G were transfected with PEI into 293T cells. Approximately 24 h post transfection the media was replaced with fresh DMEM containing 10% FBS. Viral supernatants were harvested and filtered 24 h later and immediately concentrated with a 20–24 h spin at 6000×*g*. Approximately 100× concentrated virus was stored at −80 °C.

### CRISPR–Cas9 screening

The Human GeCKOv2 whole-genome library^79^ was used in lentiviral pooled format to transduce HEL cells. For each screen replicate, cells were transduced at ~1000-fold representation of the library (at 30% infection efficiency). 2 days after transduction, 2 µg/mL puromycin was added for 3 days. A portion of cells representing 500-fold coverage of the library were harvested as the day 0 timepoint. The rest of the cells were then passaged to maintain 500-fold representation and cultured for an additional 7 days. Genomic DNA was extracted, and a two-step PCR procedure was employed to amplify sgRNA sequences and then to incorporate deep-sequencing primer sites onto sgRNA amplicons. For the first PCR, the amount of genomic DNA for each sample was calculated in order to achieve 500-fold coverage over the library and a sufficient number of PCR reactions were performed with 2 μg genomic DNA in each reaction using Herculase II Fusion DNA Polymerase (Agilent). Afterwards, a second PCR was performed to add on Illumina adapters and to barcode samples, using 5 µL of the product from the first PCR. Resulting amplicons from the second PCR were column purified using the combination of PureLink PCR purification kit (LifeTechnologies). The whole amplification was carried out with 12 cycles for the first PCR and 16 cycles for the second PCR to maintain the linear amplification. The sequences for the primers are found in Supplementary Data 8. Purified PCR products were sequenced using HiSeq 2500 (Illumina). Bowtie^80^ was to align the sequenced reads to the guides. The R/Bioconductor package edgeR^81^ was used to assess changes across various groups. Guides having a fold change >1 and an adjusted FDR < 0.05 were considered statistically significant.

### CRISPR and ribosome profiling retests

For individual gene retests of the initial whole-genome CRISPR–Cas9 screen, 4 sgRNA pools were cloned, as described above. The sgRNAs were selected by combining the sgRNAs which scored in the primary screen with additional sgRNAs from the human CRISPR Brunello lentiviral pooled library. 5 × 10^5^ HEL cells were transduced at MOI 10 and CD235a expression was measured by flow cytometry after 10 days. All sgRNA sequences can be found in Supplementary Data S8.

For translational targets from the ribosome profiling, individual retest pools for non-essential genes contained 3 sgRNAs but were otherwise cloned and tested as described above for the CRISPR–Cas9 screen. For essential genes shRNAs were selected from the Genetic Perturbation Platform database (portals.broadinstitute.org/gpp/public/) based on those with the highest adjusted score. Three shRNA were used per gene and tested individually. 5 × 10^5^ HEL cells were transduced at MOI 10 and CD235a expression was measured by flow cytometry after 4–7 days. All sgRNA and shRNA sequences can be found in Supplementary Data S8.

### METTL3 rescue of loss of CD235a expression

HEL cells were transduced with lentiCRISPRv2 containing METTL3 sgRNA sgMETTL3-S1 as described above and found in Supplementary Data 8. This particular sgRNA cuts at the 3′ splice site of exon 2, resulting in editing of the genomic sequence, but not the mRNA sequence. Four days post-transduction of CRISPR virus, the cells were transduced with either WT *METTL3* or catalytically inactive *METTL3* (395–398 DPPW to APPA)^15^ expressing virus as described above. CD235a expression was measured by flow cytometry after an additional 4–6 days. Western blotting for METTL3 expression levels was performed as described below.

### Adult bone marrow CD34+ cell isolation and culture

Bone marrow aspirates were collected under FHCRC IRB protocol 0999.209, with consent obtained for use of left-over specimens for research purposes. The material was deidentified prior to being provided for research use. The collected aspirates were washed twice in 1× PBS, Ficoll fractionated and the mononuclear cell fraction collected. Enrichment for CD34+ cells was done by magnetic bead isolation using the CD34 MicroBead Kit (Miltenyi Biotec). CD34+ HSPCs were grown in StemSpan SFEM II (Stemcell Technologies) supplemented with the following growth factor cocktails: expansion (SCF 100 ng/mL, FLT-3 ligand 10 ng/mL, TPO 100 ng/mL, IL-6 20 ng/mL), erythroid (SCF 20 ng/mL, IL-3 10 ng/mL, and EPO 4 U/mL), myeloid (SCF 100 ng/mL, FLT-3 ligand 10 ng/mL, IL-3 20 ng/mL, IL-6 20 ng/mL, GM-CSF 20 ng/mL, M-CSF 20 ng/mL, and G-CSF 20 ng/mL), megakaryocyte (SCF 10 ng/mL, IL-6 20 ng/mL, IL-9 12.5 ng/mL, and TPO 100 ng/mL). EPO was purchased from PeproTech and all other cytokines were purchased from Shenandoah Biotechnology.

### Adult bone marrow CD34+ cell transduction

CD34+ cells were thawed and cultured in StemSpan SFEM II+ expansion growth factor cocktail for 16 h prior to transduction. Plates were then coated with 10 µg/cm^2^ RetroNectin (Takara) for 2 h at RT, blocked for 30 min at RT with 2% BSA, followed by preloading with virus (final MOI 50) for 15 min at RT. hBM CD34+ cells were then cultured on the plates for 48 h at 500,000 cells/mL in StemSpan SFEM II+ expansion growth factor cocktail. The cells were then washed twice with 1× PBS and re-plated in StemSpan SFEM II+ expansion growth factor cocktail at 250,000/mL.

### Flow cytometry

HEL cells were stained with APC-CD235a (BD Pharmingen 551336). To monitor CD34+ progenitor cell differentiation status the cells were stained with PE-CF594-CD34 (BD Pharmingen 562383), APC-R700-CD38 (BD Pharmingen 564979), PE-CD123 (BD Pharmingen 554529), Pacific Blue CD45RA (Invitrogen MHCD45RA28), APC-H7-CD41 (BD Pharmingen 561422), and BV786-CD71 (BD Pharmingen 563768). To monitor lineage status cells were stained with APC-CD235a (BD Pharmingen 551336) and PE-CD71(BD Pharmingen 555537) for erythropoiesis, PE-CD14 (BD Pharmingen 555398) for myeloid cells, and PE-CD41 (BD Pharmingen 557297), and APC-CD61 (BD Pharmingen 564174) for megakaryopoiesis. Cells were analyzed on an BD LSRII or BD Symphony.

### CFU assays

10^4^ CD34+ shWTAP transduced cells were plated for lineage-specific colony assays (triplicate) in methylcellulose (MethoCult H4230, Stem Cell Technologies) supplemented with the lineage-specific growth factor cocktails outlined above. Colonies were scored after 14 days in culture.

### MeRIP-seq

MeRIP-seq was performed largely as described by Dominissini et al. ^36^. Total RNA from HEL cells was isolated by Trizol (ThermoFisher) and the Direct-zol RNA kit (Zymo Research). Poly(A) RNA was then isolated with the NucleoTrap mRNA Mini kit (Macherey–Nagel) yielding ~5 µg of polyA RNA per replicate. The polyA RNA was then fragmented using the NEBNext Magnesium RNA Fragmentation Module (NEB) for 7 min at 94 °C yielding RNA fragments of ~125 bp. Fragmented RNA was incubated with 5 µg m^6^A antibody (Millipore Sigma, ABE572) for 2 h, followed by immunoprecipitation and phenol:chloroform cleanup and ethanol precipitation. Sequencing libraries were generated using the KAPA Biosystems Stranded RNA-Seq Kit (Roche), were quantified using a Qubit Fluorometer (ThermoFisher), and the size distribution was checked using TapeStation 4200 (Agilent Technologies). 50 bp paired-end reads were sequenced on an Illumina HiSeq 2000.

### Primary cell MeRIP-seq

Total RNA from HEL cells of flow sorted primary hematopoietic cell populations was isolated by Trizol (ThermoFisher) and the Direct-zol RNA kit (Zymo Research). The concentration of total RNA was measured by Qubit RNA HS Assay Kit (ThermoFisher). A total of 3 µg of total RNA was then fragmented using the NEBNext Magnesium RNA Fragmentation Module (NEB) for 6 min at 94 °C yielding RNA fragments of ~150 bp. The fragmented RNA was precipitated overnight at −80 °C and resuspended in 10 µL H_2_O per 1ug of total RNA. Per sample 30 µL of protein-A magnetic beads (NEB) and 30 µL of protein-G magnetic beads (NEB) were washed twice with IP buffer (150 mM NaCl, 10 mM Tris-HCl, pH 7.5, 0.1% IGEPAL CA-630 in nuclease-free H_2_O), resuspended in 250 μL of IP buffer, and tumbled with 5 μg anti-m6A antibody at 4 °C for at least 2 h. Following two washes in IP buffer, the antibody-bead mixture was resuspended in 500 μL of IP reaction mixture containing the fragmented total RNA (minus 50 ng of input control RNA), 100 µL of 5 × IP buffer and 5 µL of RNasin Plus RNase Inhibitor (Promega), and incubated for 2 h at 4 °C. The RNA reaction mixture was then washed twice in 1 mL of IP buffer, twice in 1 mL of low-salt IP buffer (50 mM NaCl, 10 mM Tris-HCl, pH 7.5, 0.1% IGEPAL CA-630 in nuclease-free H_2_O), and twice in 1 mL of high-salt IP buffer (500 mM NaCl, 10 mM Tris-HCl, pH 7.5, 0.1% IGEPAL CA-630 in nuclease-free H_2_O) for 10 min each at 4 °C. After washing, the RNA was eluted from the beads in 200 μL of RLT buffer supplied in RNeasy Mini Kit (QIAGEN) for 2 min at room temperature. The magnetic separation rack was applied to pull beads to the side of the tube and the supernatant was collected to a new tube. 400 μL of 100% ethanol was added to the supernatant and RNA isolated following the manufactures instructions and eluted in 14 μL. 2 μL of eluted RNA was reverse transcribed with SuperScript IV Reverse Transcriptase (ThermoFisher) and IP efficiency was assessed by KLF1/GAPDH qPCR in a HEL control sample. 3.5 μL of eluted RNA and 50 ng of input RNA were used for library construction with the SMARTer® Stranded Total RNA-Seq Kit v2—Pico Input Mammalian (Takara) according to the manufacturer’s protocol. Libraries for IP RNA were PCR amplified for 16 cycles whereas 12 cycles were used for input RNA. The purified libraries were quantified using a Qubit Fluorometer (ThermoFisher), and the size distribution was checked using TapeStation 4200 (Agilent Technologies) and 50 bp paired-end reads were sequenced on an Illumina HiSeq 2500.

### MeRIP-qPCR

m^6^A RNA was isolated as described for primary cell meRIP-seq without the fragmentation step. Random hexamers and SuperScript IV Reverse Transcriptase (ThermoFisher) were used to generate cDNA and qPCR run with PowerSYBR Green Master Mix (ThermoFisher) on a QuantStudio 7 Flex (ThermoFisher). All primer sequences can be found in Supplementary Data S8.

### MeRIP-seq analysis

50 bp paired-end sequenced reads were mapped to hg19 using TopHat v2 (ref. ^82^) and the resulting bam files were processed using Picard tools (http://broadinstitute.github.io/picard) to mark duplicate reads. Custom R scripts were written for identification of peaks based on^37^ and for visualization of peaks. The Human transcriptome (from hg19) was broken into 25nt wide discrete non-overlapping reads. Using bedtools^83^ the 50 bp reads were mapped to 25nt windows and windows with fewer than 5 aligned reads were dropped. We used one sided Fisher’s exact test to compare the number of reads that mapped to a given window for the MeRIP sample and the non-IP sample to the total number of reads in each. The Benjamini–Hochberg procedure was used to adjust the *p*-values from the Fisher’s exact test to reduce our false discovery rate to 5%. To find a final *p*-value for each window, Fisher’s method was used to combine *p*-values across replicates. To find distinct m^6^A peaks, we combined consecutive significant 25 nt windows across the transcriptome. Consecutive significant windows <100 bp were discarded. Gene region annotations for the peaks were found from UCSC RefSeq Data for Hg19 using R/Bioconductor package TxDb.Hsapiens.UCSC.hg19.knownGene (https://bioconductor.org/packages/release/data/annotation/html/TxDb.Hsapiens.UCSC.hg19.knownGene.html). Motif calling was done using HOMER (http://homer.ucsd.edu/homer/motif/) and the default settings.

### RNA-seq and splicing analysis

HEL *sgGYPA*-KO, *sgGATA1*-KO, *sgLMO2*-KO, and *sgWTAP*-KO cells were lysed with Trizol (ThermoFisher). Total RNA was isolated with the Direct-zol RNA kit (Zymo Research) and quality validated on the Agilent 2200 TapeStation. Illumina sequencing libraries were generated with the KAPA Biosystems Stranded RNA-Seq Kit (Roche) and sequenced using HiSeq 2500 (Illumina). RNA-seq reads were aligned to the UCSC hg19 assembly using Tophat2 (ref. ^82^) and counted for gene associations against the UCSC genes database with HTSeq^84^. The normalized count data was used for subsequent Principal component analysis and Multidimensional scaling (MDS) in R. Differential Expression analysis was performed using R/Bioconductor package DESeq2 (ref. ^85^). Heatmaps were made using R/Bioconductor package pheatmap (https://CRAN.R-project.org/package = pheatmap).

The rMATS^42,86^ (http://rnaseq-mats.sourceforge.net/) pipeline version 4.0.2 was used to detect differential alternative splicing events from RNA-Seq data using the parameters -t paired --readLength 50 --cstat 0.0001. The Miso pipeline^41^ was used to analyze the RNA-seq data for alternatively spliced transcripts. First, the expression levels (psi values) were computed for each of the paired-end RNA-seq samples individually using ‘miso --run’, followed by calculating the Psi values for each sample using ‘summarise-miso’. Lastly, all pairwise comparisons between the *sgNTC* and *sgWTAP* samples were run using ‘compare_miso’ and the events were fitered using criteria ‘--num-inc 1 --num-exc 1 --num-sum-inc-exc 10 --delta-psi 0.10 --bayes-factor 10′. Only those alternative splicing events that were present in all pairwise comparisons were included in our final results.

### m^6^A colorimetric quantification

The manufactures instructions for the m^6^A RNA Methylation Quantification Kit (Abcam) were followed. HEL cells transduced with *sgWTAP, sgMETTL3, sgMETTL14,* and *sgLMO2* were flow sorted for CD235a^−^^/low^ expression. RNA was isolated as described above for RNA-seq and 200 ng of total RNA used per assay. Samples were assayed in triplicate.

### m^6^A luciferase reporters

HEL cells were transduced in triplicate with the lv-m^6^A-luciferase reporters at an MOI 10 as described above and cultured for 4 days. The cells were then collected, and half used for detection of luciferase with the Dual-Luciferase Reporter System (Promega) following the manufactures instructions and luminescence measured on a GloMax Multi + (Promega). Genomic DNA was extracted from the other half using the E.Z.N.A. MicroElute Genomic DNA Kit (Omega Bio-tek). The transduction efficiency of each sample was then quantified by WPRE qPCR as previously described^87^ (WPRE-F: GTCCTTTCCATGGCTGCTC; WPRE-R: CCGAAGGGACGTAGCAGA) using PowerSYBR Green Master Mix (ThermoFisher) on a QuantStudio 7 Flex (ThermoFisher). The luciferase signal was then normalized to the transduction efficiency.

### Ribosome profiling and analysis

The profiling methodology was based largely on protocols described by Ingolia and Hsieh and colleagues^43,44^. Wild-type and *WTAP* knockdown HEL cells were utilized to generate both ribosome protected RNA libraries as well as alkaline digested total RNA libraries using the ARTseq Ribosome Profiling Kit (Illumina). Ribo-Zero (Illumina) was used to subtract rRNA levels from each replicate. Once samples were generated, they were sequenced using an Illumina HiSeq 2500. The raw sequence data was uncompressed followed by clipping the 3′ adapter sequence (AGATCGGAAGAGCACACGTCT). Next, trimmed sequence reads were aligned to an rRNA reference using Bowtie^80^. rRNA alignments were removed to reduce rRNA contamination. The unaligned reads were collected and TopHat2 (ref. ^82^) was utilized to align non-rRNA sequencing reads to hg19. Reads for each gene were counted using HTSeq (UCSC reference transcriptome)^84^. Reads were only counted starting from 20 nucleotides after the start codon and up to 20 nucleotides before the stop codon. R/Bioconductor package, DESeq2 (ref. ^85^) was used to identify differentially expressed genes at the translational level using both ribosome bound and total RNA samples. A statistical cutoff of FDR < 0.05 and log2-fold change > 1 was applied to find translationally and transcriptionally regulated genes. RiboseqR^88^ was used to calculate triplate periodicity in all samples. Translation efficiency is a measure of ribosome bound mRNA over total mRNA and is a snapshot of the translation rate of an mRNA of interest. R/Bioconductor package, edgeR^89^ was used to find differentially expressed gene at the transcriptional level using only rRNA subtracted total RNA specimens.

### CUT&RUN and analysis

CUT&RUN was performed as described by Skene et al.^90^. 200,000 HEL cells were harvested and bound to Concanavalin A–coated beads at RT for 10 min. The bound cells were permeabilized with 0.025% Digitonin and incubated with a 1:50 dilution of H3K4me3 primary antibody (Cell Signaling Technologies), a 1:100 dilution of H3K27me3 primary antibody (Cell Signaling Technologies) as a positive control and, a 1:50 dilution of Guinea Pig anti-Rabbit IgG (Antibodies-Online) as a negative control, followed by incubation with rotation overnight at 4 °C. The antibody bound cells were incubated with the pA-MNase at a 1:10 dilution for 1 h at 4 °C, the MNase digestion was then run for 30 min at 0 °C and then incubated at for 10 min at 37 °C to release the DNA fragments. Released DNA fragments were extracted using the NucleoSpin Gel and PCR Clean-up kit (Macherey–Nagel) and sequencing libraries generated using the KAPA HyperPlus kit (Roche) following the manufactures instructions.

Sequencing was performed using an Illumina HiSeq 2500 in Rapid Run mode and employed a paired-end, 50 bp read length (PE50) sequencing strategy. Raw fastq files were aligned with Bowtie^80^ to the human genome (hg19) and spike in to the *Saccharomyces cerevisiae* genome (sacCer3), followed by spike in calibration. Picard (https://broadinstitute.github.io/picard/) was used to remove duplicate reads and aligned fragments were extracted from the bam file to a bed file. The bed file was used to call peaks following the protocol described by Skene et al.^90^. EaSeq was used for data visualization^91^.

### Actinomycin D mRNA stability assay

HEL cells were transduced with NTC, METTL3, METTL14, and WTAP sgRNAs as described above. 8 days post-transduction, reduced CD235a expression was verified by flow cytometry as described above and the HEL cells were treated with 10 µg/mL of Actinomycin D. Cells were harvested in Trizol at 0, 2, 4, 6, and 8 h following addition of Actinomycin D and total RNA was isolated using the Direct-zol RNA kit (Zymo Research). The mRNA levels of the target genes were quantified by qPCR, using PowerSYBR Green Master Mix (ThermoFisher) on a QuantStudio 7 Flex (ThermoFisher) and compared to expression at the 0 h timepoint.

### Chromatin immunoprecipitation

Chromatin immunoprecipitation was performed using the SimpleChIP Plus Sonication Chip Kit (Cell Signaling Technology #56383), following the manufactures instructions. HEL cells were cross-linked with 0.4% formaldehyde for 10 min. 1 × 10^7^ cells were used for each immunoprecipitation with 3 μg of KLF1 antibody (Abcam ab2483) or IgG (Abcam ab37373). Quantification was done using PowerSYBR Green PCR Master Mix (Applied Biosystems) on an Applied Biosystems QuantStudio 7 Flex real-time PCR machine, according to the manufacturer’s instructions. Primer sequences are listed in Supplementary Data 8.

### Western blot and puromycin incorporation

Immunoblots were performed following standard protocols (www.cshprotocols.org). HEL cells were lysed in a modified RIPA buffer (150 mM NaCl, 50 mM Tris, pH 7.5, 2 mM MgCl_2_, 0.1% SDS, 2 mM DDT, 0.4% Triton X-100), 1× complete protease inhibitor cocktail (complete Mini EDTA-free, Roche) on ice for 15 min. Cell lysates were quantified using the Pierce BCA Protein Assay Kit (ThermoFisher). The Trans-Blot Turbo transfer system was used according to the manufacturer’s instructions. The following commercial antibodies were used: CXXC1 (Cell Signal, 1:250), PABPC4 (Novus Biologicals, 1:500), PABPC1 (ThermoFisher, 1:500), WTAP (Abcam, 1:500), BRD7 (ThermoFisher, 1:250), STK40 (ThermoFisher, 1:250), TADA2B (Abnova, 1:250), Beta-actin (Cell Signaling, 1:1000), and Beta-tubulin (Abcam, 1:1000). An Odyssey infrared imaging system (LI-COR) was used to visualize blots following the manufacturer’s instructions. The Image Studio software was used to semi-quantify the blots.

The puromycin incorporation assay was performed as previously reported^92^. HEL cells were transduced with *sgNTC* or sg*WTAP* and cultured for 10 day. On day 10, the cells were treated with 10 µg/mL of puromycin for 10 min at 37 °C. Immunoblotting for puromycin incorporation (Millipore Sigma, 1:1000) was done as described above.

### Single-cell western blotting

Adult bone marrow CD34+ HSPCs were transduced with *shScr* or *shWTAP* lentivirus and cultured in the expansion growth factor cocktail as described above. Five days post transduction the cells were flow sorted for the transduced (GFP+) MEP progenitor population as shown in Fig. 6g. The sorted cells were then immediately loaded onto the Milo small scWest Chip (ProteinSimple) following the manufactures instructions^93^. The following run conditions were used: lysis—5 s; electrophoresis—70 s; UV capture 4 min. The chips were probed with the following antibodies: PABPC4 (Novus Biologicals, 1:20), PABPC1 (ThermoFisher, 1:20), WTAP (Abcam, 1:20), Beta-tubulin (Abcam, 1:20) and Alexa Fluor 555 donkey anti-rabbit (Invitrogen, 1:40) was used for the secondary antibody. Chips were scanned on a GenePix 4000B (Molecular Devices) and analyzed with the scout software package (ProteinSimple).

### Gene set enrichment analysis

Genes sets arising from our genomic datasets were analyzed using GSEA^94^, the ToppGene tool suite^95^, or GeneMANIA network viewer plugin for Cytoscape^96,97^.

### Reporting Summary

Further information on research design is available in the Nature Research Reporting Summary linked to this article.

## Supplementary information

Supplementary Information

Reporting Summary

Description of Additional Supplementary Files

Supplementary Data 1

Supplementary Data 2

Supplementary Data 3

Supplementary Data 4

Supplementary Data 5

Supplementary Data 6

Supplementary Data 7

Supplementary Data 8

Source Data

## Data Availability

A reporting summary for this article is available as a Supplementary Information File. sgRNA-seq, RNA-seq, Ribo-seq, CUT&RUN and MeRIP-seq raw reads and processed datasets can be accessed in the NCBI Gene Expression Omnibus under accession number GEO: GSE106124. The HEK293T cell MeRIP-seq data was published by Meyer et al.^37^ and can be accessed in the NCBI Gene Expression Omnibus under accession number GEO: GSE29714. The source data underlying Figs. 1f and 4d and Supplementary Figs. 3a and 5f are provided as a Source Data file. All other data are available from the corresponding author P.J.P. on reasonable request.

## Code availability

m^6^A peak calling was performed using custom R scripts that will be provided upon request. All other analyses were performed using publicly available software as indicated.
