# Supplementary material for: N6-methyladenosine mRNA marking promotes selective translation of regulons required for human erythropoiesis
Source: Nat Commun. 2019 Oct 10;10:4596. doi: 10.1038/s41467-019-12518-6 (PMC6787028; doi:10.1038/s41467-019-12518-6)
Supplement: Supplementary file 1 — Supplementary Information [file 41467_2019_12518_MOESM1_ESM.pdf]

N^6-methyladenosine mRNA marking promotes selective translation of regulons required for human erythropoiesis

Kuppers et al.

Supplementary Information

Supplementary Fig. 1

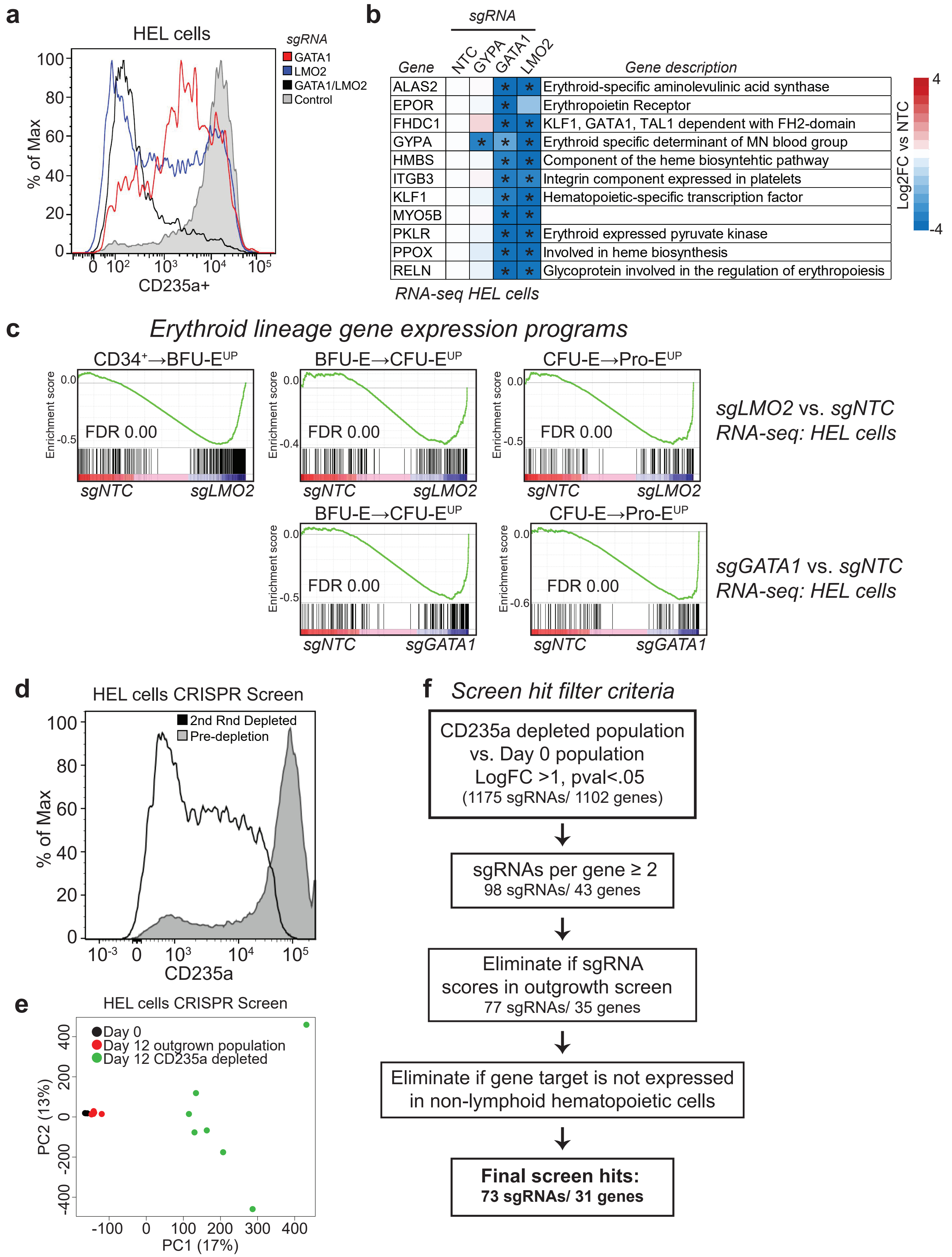

Supplementary Fig. 1: Validation of HEL cells as a surrogate model of erythropoiesis and whole genome CRISPR screening a, Flow cytometry for CD235a expression in HEL cells 10 days post-transduction with lv-sgRNA-KO for *LMO2*, *GATA1* or the two combined. **b,** Gene expression changes for select GATA1 transcriptional targets (as defined by Yu et al.[1]) in HEL cells following *sgLMO2*-KO or *sgGATA1*-KO as quantified by RNA-seq (n=3). *Indicates significant changes relative to non-targeting control (sgNTC) (FDR<.05). **c,** Gene expression changes in HEL cells following *GATA1*-KO or *LMO2*-KO are negatively correlated with erythroid stage-specific genes up-regulated during normal erythropoiesis by GSEA analysis (detailed in Methods). These gene sets can be found in (Supplementary Data 7). **d,** A representative example of HEL cell enrichment, on day 12 post-transduction with the whole genome CRISPR-Cas9 library, for CD235a-/low cells following two rounds of magnetic bead depletion of CD235a high cells (see Methods for details). **e,** Principal component analysis for altered sgRNA representation in the seven replicates of Day 12 post-transduction CD235a-/low HEL cells, as shown in Fig. 1a, versus all outgrown cells at Day 12 post-transduction and the Day 0 samples (Day 5 post-transduction) for the initial representation of sgRNAs within the transduced cells (n=7). **f,** The filter criteria used to define the screen hits arising in the CD235a-/low population and retested in Fig. 1b.

Supplementary Fig. 2

a

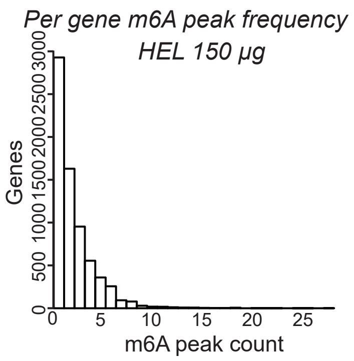

b

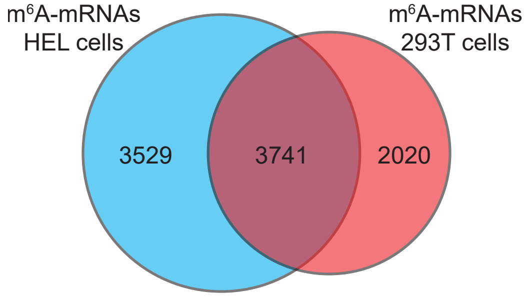

c Top 5 GO: Human Phenotype - Shared 293T and HEL unique m^{6}A-mRNAs

ID	Name	FDR B&H	Genes from Input	Genes in Annotation
HP:0000252	Microcephaly	1.52E-12	223	766
HP:0000347	Micrognathia	3.65E-11	166	536
HP:0001263	Global developmental delay	1.01E-09	354	1454
HP:0001249	Intellectual disability	4.92E-08	317	1305
HP:0000431	Wide nasal bridge	1.07E-07	118	378

d

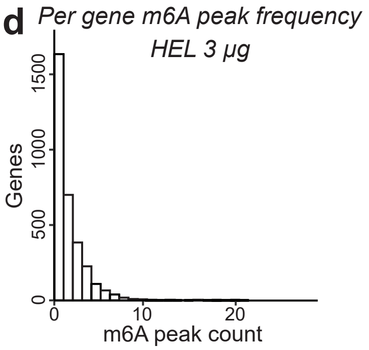

e

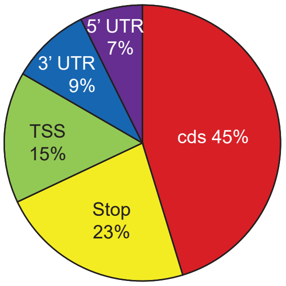

f

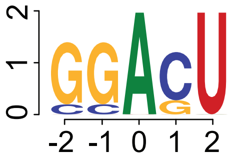

g

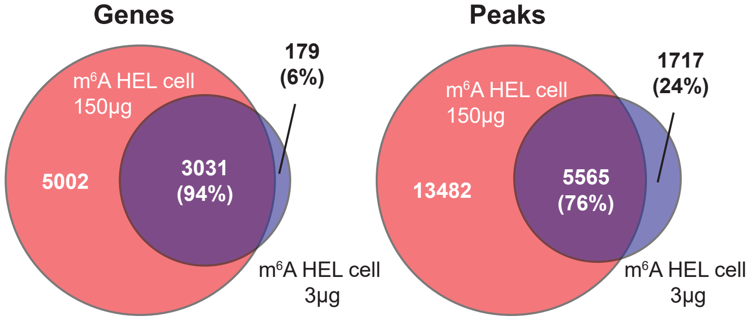

h

Sample	Lineage Marker	Unique m6A genes	Total m6A genes	% overlap HEL
All		413	2060	78.1
HSC	lin-CD38-CD34+	113	674	81.8
CMP	CD34+CD38+IL-3Rα^{lo}+CD45RA-	76	815	88.1
GMP	CD34+CD38+IL-3Rα^{lo}+CD45RA+	55	326	81
CD14	FSC^{hi}SCC^{low}CD14+	232	928	74.1
MEP	CD34+CD38+IL-3Rα^{lo}-CD45RA-	37	268	81.1
MEG	CD34-CD61+	87	391	77.2
ERY1	CD34+CD71+CD235a-	45	869	92.3
ERY2	CD34-CD71+CD235a-	42	357	84
ERY3	CD34-CD71+CD235a+	74	213	63.9

Supplementary Fig. 2: meRIP-seq in HEL cells and human adult BM cells

a, A histogram of the per gene frequency of m^6A peaks in methylated genes from HEL meRIP-seq using 150 µg of total RNA (HEL 150 µg). **b,** Venn diagram of m^6A mRNAs identified by meRIP-seq in 293T cells[2] and in HEL 150 µg cells. **c,** ToppGene GO analysis of m^6A genes overlapping the HEL 150 µg and 293T[2] data sets. **d,** A histogram of the per gene frequency of m^6A peaks in methylated genes from HEL 3 µg, showing a similar pattern to HEL 150 µg meRIP-seq data (compare to Supplementary Fig. 2a). **e,** Pie chart displaying the frequency of m^6A peaks, from HEL 3 µg, within different transcript regions: TSS, centered around translation start ATG, Stop, centered around the stop codon. The distribution is consistent with the HEL 150 µg data (Fig. 2b) **f,** The enriched m^6A methylation site motif detected by meRIP-seq in HEL cells using 3 µg of total RNA (HEL 3 µg). **g,** Venn diagrams for m^6A mRNAs and peaks identified by MeRIP-seq in HEL 150 µg and in HEL 3 µg cells, showing significant agreement between the data sets, but clear undersampling in the HEL 3 µg data. **h,** A summary of the surface marker criteria used to isolate hematopoietic populations from adult human BM for meRIP-seq, as well as the total number of m^6A containing genes identified for each population and the number of unique methylated genes detected in each population. Supplementary Data 2 contains the complete data set.

Supplementary Fig. 3

a

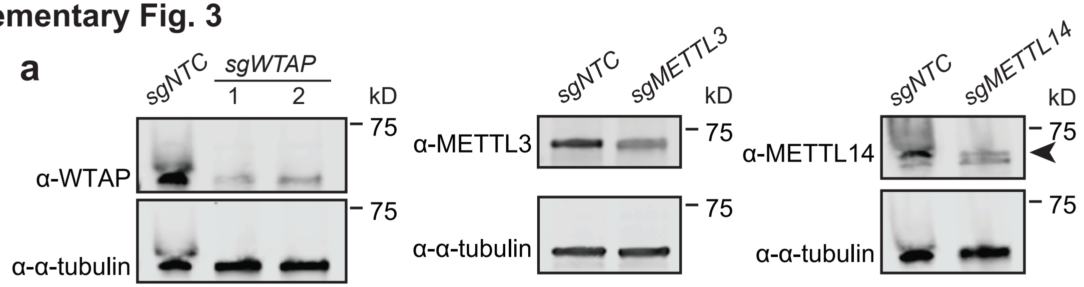

b

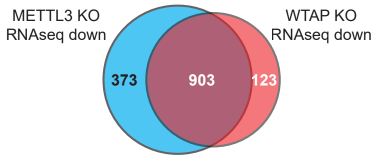

c

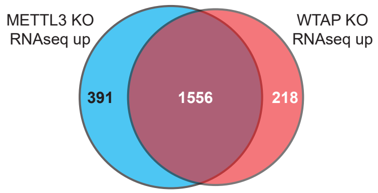

GO: Molecular Function

ID	Name	p-Value	FDR B&H	Genes from input	Genes in Annotation
GO:0005344	oxygen carrier activity	6.01E-07	9.36E-04	7	14
GO:0008201	heparin binding	7.38E-06	5.74E-03	21	167

GO: Biological Process

ID	Name	p-Value	FDR B&H	Genes from input	Genes in Annotation
GO:0009205	purine ribonucleoside triphosphate metabolic process	1.32E-12	7.28E-09	40	263

Coexpression Atlas

ID	Name	p-Value	FDR B&H	Genes from input	Genes in Annotation
PP MEP 1000 K1	PP MEP top-relative-expression-ranked 1000 k-means-cluster#1	3.49E-16	2.70E-12	34	161
PP RBC 500 K2	PP RBC top-relative-expression-ranked 500 k-means-cluster#2	3.64E-15	1.17E-11	38	216

GO: Molecular Function

ID	Name	p-Value	FDR B&H	Genes from input	Genes in Annotation
GO:0005102	signaling receptor binding	3.81E-11	7.28E-08	181	1601
GO:0003700	DNA-binding transcription factor activity	4.46E-07	1.70E-04	135	1264
GO:0019955	cytokine binding	5.57E-07	1.78E-04	23	103
GO:0032395	MHC class II receptor activity	4.79E-06	1.31E-03	7	12

GO: Biological Process

ID	Name	p-Value	FDR B&H	Genes from input	Genes in Annotation
GO:0006955	immune response	2.31E-22	1.72E-18	214	1572
GO:0007155	cell adhesion	1.42E-15	2.64E-12	190	1530
GO:0050776	regulation of immune response	3.07E-14	4.56E-11	126	899

Coexpression Atlas

ID	Name	p-Value	FDR B&H	Genes from input	Genes in Annotation
PP GMP 100	PP GMP top-relative-expression-ranked 1000	1.02E-34	8.28E-31	174	987
GSM854273	Myeloid Cells, DC.103-11b+F4/80lo.Kd, CD45+ CD11c+ I-A/I-E+ CD11b+ F4/80lo, Kidney, avg-3	1.94E-33	7.83E-30	102	406
GSM854326	Myeloid Cells, MF.Microglia.CNS, CD45low CD11b+ F4/80+, Brain, avg-3	1.50E-30	2.43E-27	98	407

d

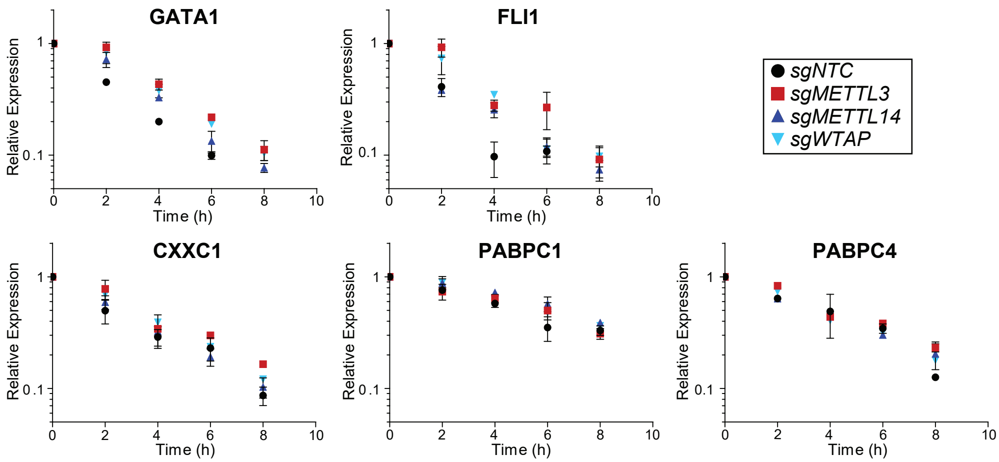

e

MATS

m6A associated alternative splicing with *WTAP* KO

Splicing Group	No of events in splicing group	Overlap of m6A site & splicing group	Hypergeometric test p-value	Unique m6A genes
All Groups	5045	1018	1	708
a3ss	543	97	1	87
a5ss	362	83	0.999961334	74
mxe	492	111	0.99990771	96
se	2929	614	1	473
ri	719	113	1	101

m6A associated alternative splicing with *METTL3* KO

Splicing Group	No of events in splicing group	Overlap of m6A site & splicing group	Hypergeometric test p-value	Unique m6A genes
All Groups	5110	1086	1	739
a3ss	533	91	1	82
a5ss	381	89	0.999690865	81
mxe	541	135	0.998453052	112
se	3001	663	1	496
ri	654	108	1	95

MISO

m6A associated alternative splicing with *WTAP* KO

Splicing Group	No of events in splicing group	Overlap of m6A site & splicing group	Hypergeometric test p-value	Unique m6A genes
All Groups	337	141	0.444642995	106
a3ss	37	17	0.340500032	16
a5ss	25	11	0.468211377	11
mxe	50	25	0.135307379	20
se	137	53	0.294689426	43
ri	88	35	0.427583431	28

m6A associated alternative splicing with *METTL3* KO

Splicing Group	No of events in splicing group	Overlap of m6A site & splicing group	Hypergeometric test p-value	Unique m6A genes
All Groups	453	186	0.473269073	141
a3ss	51	21	0.550968289	20
a5ss	41	13	0.136515773	12
mxe	77	37	0.139280956	27
se	196	84	0.356746685	69
ri	88	31	0.145129112	23

Supplementary Fig. 3: *WTAP*-KO in HEL cells disrupts the erythroid transcriptional program but does not affect splicing in cis a, Western blot validation of *WTAP*-KO, *METTL3*-KO and *METTL14*-KO in HEL cells by CRISPR-Cas9. Cell were transduced with lentiCRISPRv2 as either single sgRNAs (WTAP) or pools of 3 sgRNAs (METTL3 and METTL14), as described in the Methods. Source data are provided as a Source Data file. **b**, Venn diagram defining a core profile of up regulated transcripts following *METTL3*-KO and *WTAP*-KO in HEL cells. Top GO terms for the core transcripts up regulated in HEL cells following m^6A loss. Supplementary Data 3 contains the complete results. **c**, Venn diagram defining a core profile of down regulated transcripts following *METTL3*-KO and *WTAP*-KO in HEL cells. Top GO terms for the core transcripts down regulated in HEL cells following m^6A loss. Supplementary Data 3 contains the complete results. **d,** Actinomycin D mRNA stability results. Plots of qPCR quantified mRNA levels relative to pre-treatment. None of the genes showed a significant difference in mRNA half-life between sgNTC and sgKO samples. (mean ± SEM, Student's t-test) **e,** Summary of splicing analysis of sg*WTAP*-KO and sg*METTL3*-KO HEL for enrichment in m^6A genes utilizing MISO and MATS. No significant enrichment was observed for any splicing event type. Supplementary Data 4 contains the complete data set. (n=4, *WTAP*- and *METTL3*-KO included splicing events were present in at least 6 pair-wise comparisons for MISO, Hypergeometic test)

Supplementary Fig. 4

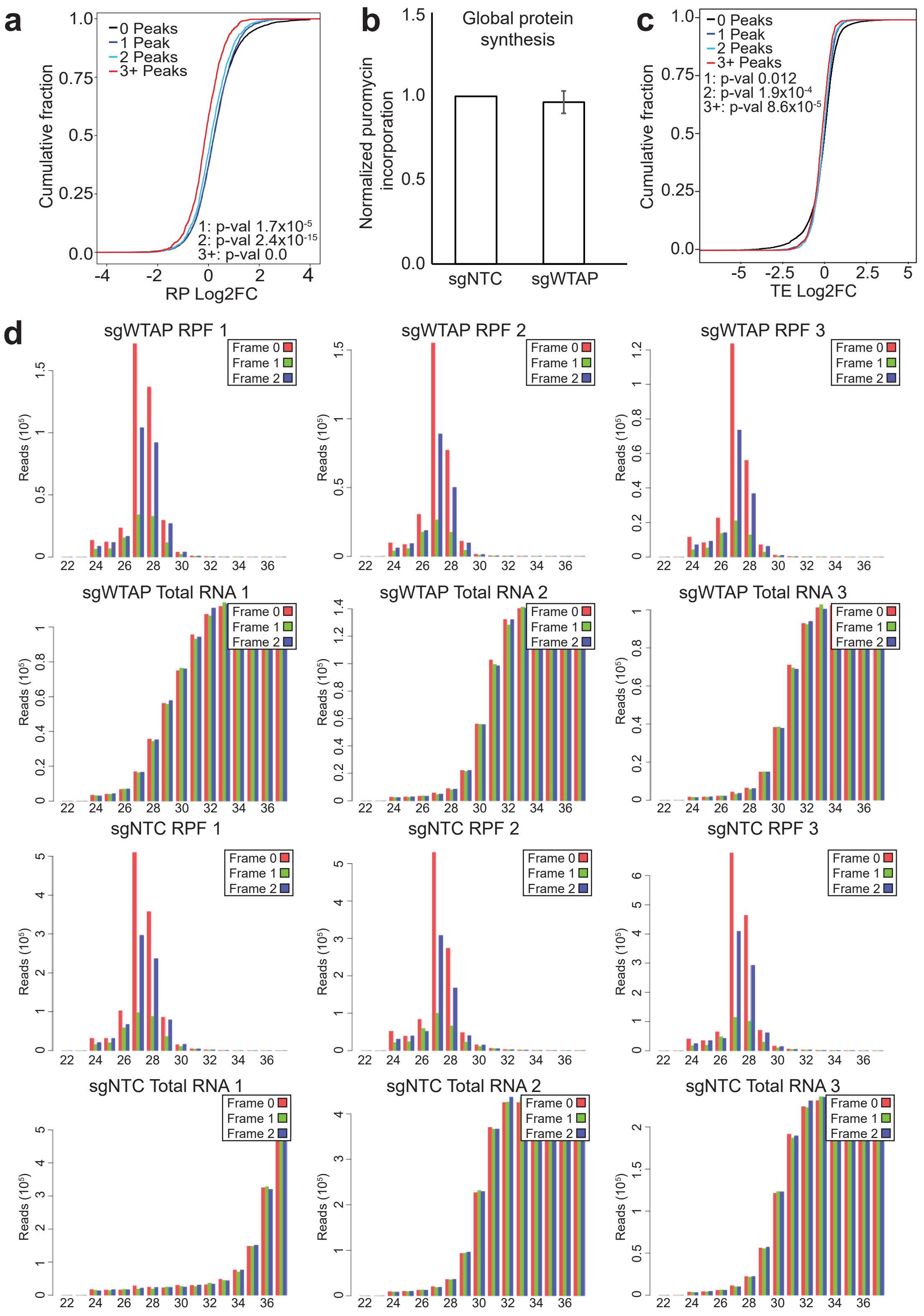

Supplementary Fig. 4: Translational changes following *WTAP*-KO a, Cumulative distribution plots, separated based on the number of m^6A peaks within a transcript, for changes in the frequency of ribosome binding in *sgWTAP*-KO compared to *sgNTC* HEL cells. A leftward-shift indicates reduced translation (n=3, Kolmogorov-Smirnov, 1-peak: P value $1.7x10^{-5}$; 2-peaks: P value $2.4x10^{-15}$; 3+peaks: P value < $1.0x10^{-20}$). **b,** Global protein synthesis quantified by Western blot for puromycin incorporation in *sgWTAP*-KO HEL cells compared to sgNTC HEL cells. No significant change was observed (n=3, s.e., P value = 0.695, t-test). **c,** Cumulative distribution plots, separated based on the number of m^6A peaks within a transcript, for changes in translational efficiency (TE) in *sgWTAP*-KO HEL cells compared to *sgNTC* HEL cells (n=3, Kolmogorov-Smirnov, 1-peak: p-value 0.012; 2-peaks: p-value 1.9x10-4; 3+peaks: p-value 8.6x10-5). **d,** Periodicity plots for *sgWTAP* and *sgNTC* HEL cells ribosome protected fraction (RPF) and RNA input sample replicates.

Supplementary Fig. 5

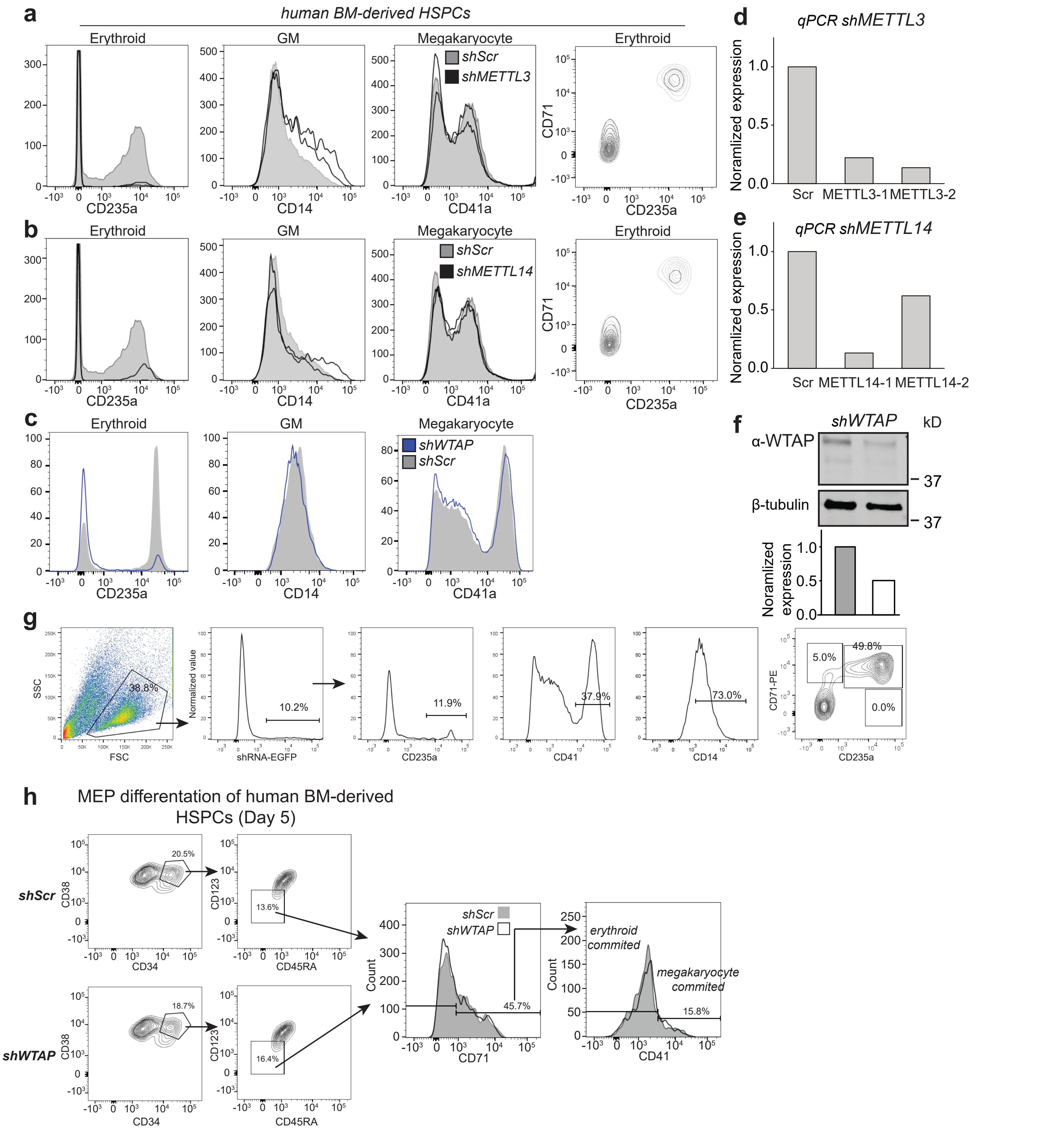

Supplementary Fig. 5: Characterization of *WTAP*-KD, *METTL3*-KD and *METTL14*-KD in HSPCs a, Flow cytometry of *METTL3*-KD hBM HSPCs differentiated in liquid culture reveals a block to erythropoiesis with no impact on megakaryopoiesis (n=3, 54.9±3.4 vs. 52.5±2.9) and an increase in myelopoiesis (n=3, 52.3±5.0 vs. 37.5±1.8). **b,** Flow cytometry of *METTL14*-KD hBM HSPCs differentiated in liquid culture reveals a block to erythropoiesis with no impact on megakaryopoiesis (n=3, 54.2±0.9 vs. 52.5±2.9) or myelopoiesis (n=3, 37.6±6.3 vs. 37.5±1.8). **c,** Flow cytometry of *WTAP*-KD hBM HSPCs differentiated in liquid culture reveals a block to erythropoiesis with no impact on megakaryopoiesis (n=3, 55.5±4.0 vs. 57.7±6.5) or myelopoiesis (n=3, 93.3±1.5 vs. 89.6±2.6). **d,** Quantification by qPCR of lv-*shMETTL3*-KD in HEL cells for two unique shRNAs. **e,** Quantification by qPCR of lv-*shMETTL14*-KD in HEL cells for two unique shRNAs. **f,** Western blot validation in HEL cells of lv-*shWTAP*-KD. Quantification is normalized to β-actin. Source data are provided as a Source Data file. **g,** Representative gating for Fig. 6a,b. **h,** Representative flow cytometry of the lineage committed progenitors within the MEP population of cultured hBM CD34+ HSPCs transduced with lv-*shWTAP* or lv-shNTC and cultured in 4-factor cocktail for 5 days shows no impact of *WTAP*-KD on lineage choice at the MEP stage. Megakaryocyte progenitors are defined as being CD41+ while erythroid progenitors are CD41- (n=3).

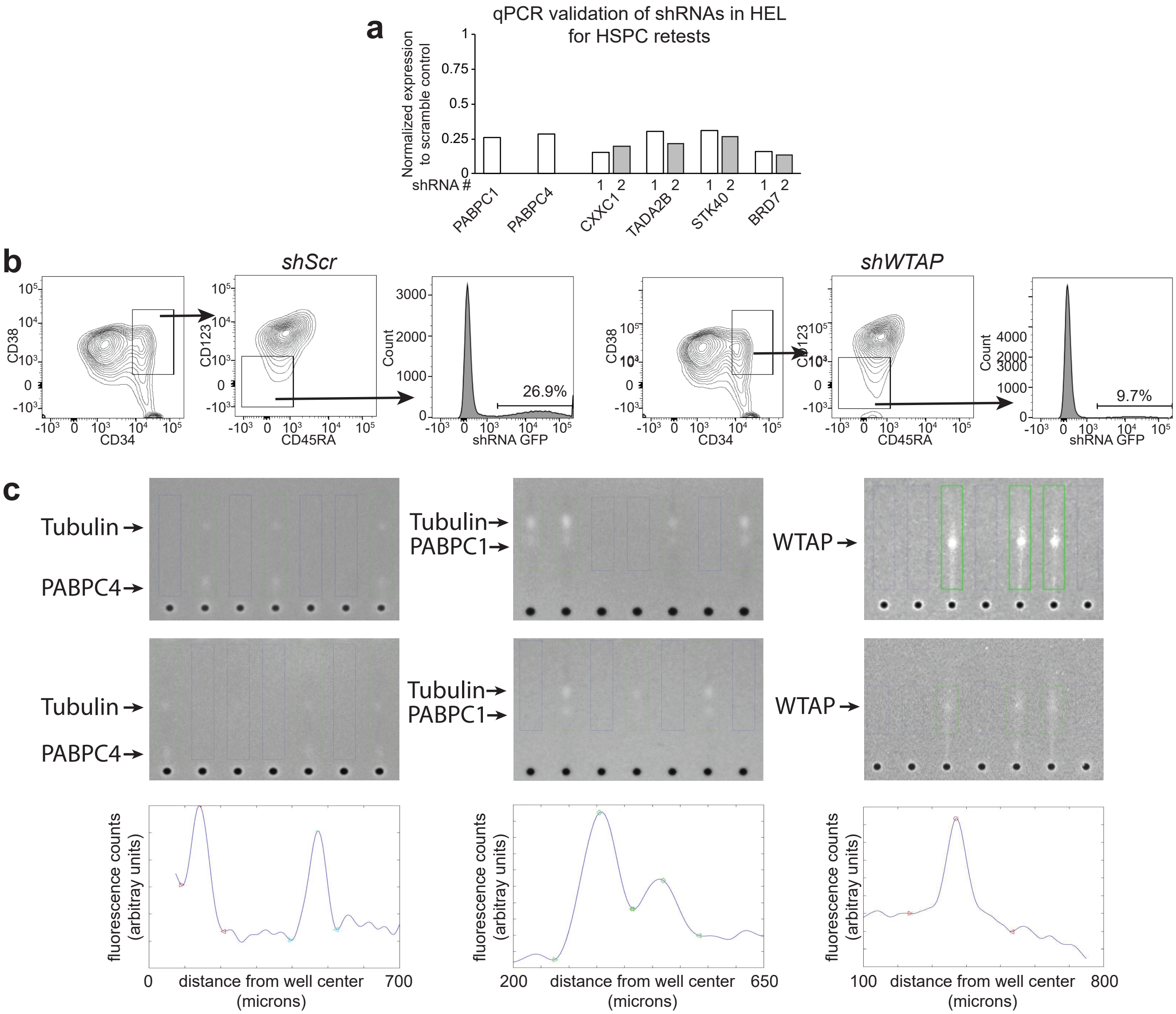

Supplementary Fig. 6 m⁶A regulation of erythropoiesis. **a,** Quantification by qPCR of lv-shRNA-KD in HEL cells 4 day post-transduction for one or two shRNAs targeting the nonessential m6A translational targets retested in Fig. 6e-g. (n=1) **b,** The flow cytometry sorting profile for MEP from shScr or shWTAP transduced hBM CD34+ HSPCs cultured for 5 days and used for single cell Western blot (scWB) analysis in Fig. 6g. **c,** Representative lanes and signal intensities on the scWB chip for WTAP, PABPC1, PABPC4 and α-Tubulin. Plots for representative peak calling using the Scout software are shown.

Supplementary References

1. Yu, M. *et al.* Insights into GATA-1-mediated gene activation versus repression via genome-wide chromatin occupancy analysis. *Molecular cell* **36**, 682-695 (2009).
2. Meyer, K.D. *et al.* Comprehensive analysis of mRNA methylation reveals enrichment in 3' UTRs and near stop codons. *Cell* **149**, 1635-1646 (2012).
