# Supplementary material for: N6-methyladenosine mRNA marking promotes selective translation of regulons required for human erythropoiesis
Source: Nat Commun. 2019 Oct 10;10:4596. doi: 10.1038/s41467-019-12518-6 (PMC6787028; doi:10.1038/s41467-019-12518-6)
Supplement: Supplementary file 2 — Reporting Summary [file 41467_2019_12518_MOESM2_ESM.pdf]

nature research

Corresponding author(s): Patrick J Paddison

Last updated by author(s): Aug 15, 2019

Reporting Summary

Nature Research wishes to improve the reproducibility of the work that we publish. This form provides structure for consistency and transparency in reporting. For further information on Nature Research policies, see Authors & Referees and the Editorial Policy Checklist .

Statistics

For all statistical analyses, confirm that the following items are present in the figure legend, table legend, main text, or Methods section.

n/a	Confirmed	
☐	☒	The exact sample size (*n*) for each experimental group/condition, given as a discrete number and unit of measurement
☐	☒	A statement on whether measurements were taken from distinct samples or whether the same sample was measured repeatedly
☐	☒	The statistical test(s) used AND whether they are one- or two-sided<br>*Only common tests should be described solely by name; describe more complex techniques in the Methods section.*
☒	☐	A description of all covariates tested
☒	☐	A description of any assumptions or corrections, such as tests of normality and adjustment for multiple comparisons
☐	☒	A full description of the statistical parameters including central tendency (e.g. means) or other basic estimates (e.g. regression coefficient) AND variation (e.g. standard deviation) or associated estimates of uncertainty (e.g. confidence intervals)
☐	☒	For null hypothesis testing, the test statistic (e.g. *F*, *t*, *r*) with confidence intervals, effect sizes, degrees of freedom and *P* value noted<br>*Give P values as exact values whenever suitable.*
☒	☐	For Bayesian analysis, information on the choice of priors and Markov chain Monte Carlo settings
☒	☐	For hierarchical and complex designs, identification of the appropriate level for tests and full reporting of outcomes
☒	☐	Estimates of effect sizes (e.g. Cohen's *d*, Pearson's *r*), indicating how they were calculated

Our web collection on statistics for biologists contains articles on many of the points above.

Software and code

Policy information about availability of computer code

Data collection	FACS Diva Software version 8.0.1,
Data analysis	The custom R scripts used for m6A peak calling will be provided upon request. All other software used is as follows: GSEA v3.0, ToppGene tool suite, Cytoscape v3.6.1, GeneMANIA v3.5.0, DESeq2 v1.20.0, Bowtie v1.2.2, TopHat v2.1.0, HTSeq v0.9.1, RiboseqR v1.14.0, edgeR v3.22.3, Miso v0.5.4, Picard v2.17.1, bedtools v2.27.0, R/Bioconductor package TxDb.Hsapiens.UCSC.hg19.knownGene v3.2.2, HOMER v4.4, EaSeq v1.05, CUT&RUN v1.0, FloJo v10.4.2, Prism v7.03, Scout v2.0

For manuscripts utilizing custom algorithms or software that are central to the research but not yet described in published literature, software must be made available to editors/reviewers. We strongly encourage code deposition in a community repository (e.g. GitHub). See the Nature Research guidelines for submitting code & software for further information.

Data

Policy information about availability of data

All manuscripts must include a data availability statement. This statement should provide the following information, where applicable:

- Accession codes, unique identifiers, or web links for publicly available datasets
- A list of figures that have associated raw data
- A description of any restrictions on data availability

sgRNA-seq, MeRIP-seq, RNA-seq, Ribo-seq, and CUT&RUN raw reads and processed data sets can be accessed in the NCBI Gene Expression Omnibus under accession number GEO: GSE106124. The HEK293T cell MeRIP-seq data was published by Meyer et al.37 and can be accessed in the NCBI Gene Expression Omnibus under accession number GEO: GSE29714. The source data underlying Figs 1f, 4d and Supplementary Figs 3a and 5f are provided as a Source Data file. All other data are available from the corresponding author P.J.P. on reasonable request.

Field-specific reporting

Please select the one below that is the best fit for your research. If you are not sure, read the appropriate sections before making your selection.

- [x] Life sciences
- [ ] Behavioural & social sciences
- [ ] Ecological, evolutionary & environmental sciences

For a reference copy of the document with all sections, see nature.com/documents/nr-reporting-summary-flat.pdf

Life sciences study design

All studies must disclose on these points even when the disclosure is negative.

Sample size	No sample size calculation was performed; sample size was based on the observed variability between independent experimental replicates with >= 2 replicates per sample. For single cell experiments a minimum of 500 cells were captured per condition.
Data exclusions	no data points were excluded
Replication	All validation results for the CRISPR-Cas9 screen were reproducible. All functional experiments were performed in replicate and the results were reliably reproduced. The scWB experiment was done once, with cells that reproduced the observed flow cytometry phenotypes.
Randomization	Not applicable, no animal studies or human research participants were involved. The CD34+ cells were isolated from a random sampling of allogenic transplant donors without bias for age or sex.
Blinding	Not applicable, no animal studies or human research participants were involved.

Reporting for specific materials, systems and methods

We require information from authors about some types of materials, experimental systems and methods used in many studies. Here, indicate whether each material, system or method listed is relevant to your study. If you are not sure if a list item applies to your research, read the appropriate section before selecting a response.

Materials & experimental systems

n/a	Involved in the study	
[ ]	[x]	Antibodies
[ ]	[x]	Eukaryotic cell lines
[x]	[ ]	Palaeontology
[x]	[ ]	Animals and other organisms
[x]	[ ]	Human research participants
[x]	[ ]	Clinical data

Methods

n/a	Involved in the study	
[x]	[ ]	ChIP-seq
[ ]	[x]	Flow cytometry
[x]	[ ]	MRI-based neuroimaging

Antibodies

Antibodies used

Flow Cytometry : All antibodies were used at 1:100 except CD235a 1:200.

BB515 Mouse Anti-Human CD61 (VI-PL2, BD Bioscience, Cat# 565123), APC Mouse Anti-Human Anti-CD235a (GA-R2, BD Bioscience, Cat# 551336), APC-H7 Mouse Anti-Human CD41a (HIP8, BD Bioscience, Cat# 561422), PE Mouse Anti-Human CD41a (HIP8, BD Bioscience, Cat# 557297), APC-R700 Mouse Anti-Human CD38 (HIT2, BD Bioscience, Cat# 564979), PE-CF594 Mouse Anti-Human CD34 (581, BD Bioscience, Cat# 562383), PE Mouse Anti-Human CD123 (7G3, BD Bioscience, Cat# 554529), Pacific Blue Anti-CD45RA (MEM-56, Thermo Fisher, Cat# MHCD45RA28), PE Mouse Anti-Human CD14 (M5E2, BD Bioscience, Cat# 555398), BV786 Mouse Anti-Human CD71 (M-A712, BD Bioscience, Cat# 563768), PE Mouse Anti-Human CD71 (M-A712, BD Bioscience, Cat# 555537), PE-Cy7 Mouse Anti-Human CD90 (5E10, BD Bioscience, Cat# 561558), Unconjugated Anti-CD235a used for CRISPR screen (GA-R2, BD Bioscience, Cat# 555569), APC Mouse Anti-Human CD61 (VI-PL2, BD Bioscience, Cat# 564174)

Western blot and scWestern blot, CUT&RUN, ChIP-qPCR and meRIP:

CXXC1 (Cell Signal, # 12585, 1:250), PABPC4 (Novus Biologicals, # NB100-74593, 1:500, scWB 1:20), PABPC1 (ThermoFisher, # PIPA529883, 1:500, scWB 1:20), WTAP (Abcam, # ab195380, 1:500, scWB 1:20), BRD7 (Invitrogen, # PA5-41544, 1:250), STK40 (Invitrogen, # PA5-26629, 1:250), TADA2B (Abnova, # H00093624-M08, 1:250), β-Actin (Cell Signaling, #3700, 1:1,000), and β-Tubulin (Abcam, # ab6046, 1:1,000, scWB 1:20). H3K4me3Tri-Methyl-Histone H3 (Lys4) used for CUT&RUN (C42D8, Cell Signaling Technology, Cat# 9751, 1:50), Tri-Methyl-Histone H3 (Lys27) used for CUT&RUN (C36B11, Cell Signaling Technology, Cat# 9733, 1:100), Guinea Pig anti-Rabbit IgG used for CUT&RUN (Antibodies-Online, Cat# ABIN101961, 1:50), Alexa Fluor 555 donkey anti-rabbit (Thermo Fisher, Cat# A31572, 1:40), N6-methyladenosine (m6A) used for meRIPseq, (Millipore Sigma, Cat# ABE572), Anti-Puromycin (12D10, Millipore Sigma, Cat# MABE343, 1:200). KLF1 (Abcam ab2483) or IgG (Abcam ab37373) used for ChIP-qPCR.

Validation

All antibodies are commercially available with validation data provided by the manufacturer. The antibodies for the scWestern blots were validated by standard WB to ensure the presence of a single band.

Eukaryotic cell lines

Policy information about cell lines

Cell line source(s)	The HEL cells were acquired from the Papayannopoulou lab (Univ. of Washington), who initially established the line. HEK293T cells were obtained from ATCC.
Authentication	The cell lines were not authenticated. The RNAseq data for the HEL cells is consistent with the described origin and cell type for the line.
Mycoplasma contamination	The HEL were confirmed to be negative for mycoplasma.
Commonly misidentified lines (See ICLAC register)	No commonly misidentified cell lines were used.

Flow Cytometry

Plots

Confirm that:

- [x] The axis labels state the marker and fluorochrome used (e.g. CD4-FITC).
- [x] The axis scales are clearly visible. Include numbers along axes only for bottom left plot of group (a 'group' is an analysis of identical markers).
- [x] All plots are contour plots with outliers or pseudocolor plots.
- [x] A numerical value for number of cells or percentage (with statistics) is provided.

Methodology

Sample preparation	Bone marrow aspirates were collected under FHCRC IRB protocol 0999.209. The cells isolated from aspirates were considered non-human subjects as no identifiable information was associated with the leftover specimen. The collected aspirates were washed twice in 1x PBS, Ficoll fractionated and the mononuclear cell fraction collected. Enrichment for CD34+ cells was done by magnetic bead isolation using the CD34 MicroBead Kit (Miltenyi Biotec). HEL cells were washed in FACS buffer(DPBS + 0.2% BSA) and collected by centrifugation prior to staining.
Instrument	BD LSRII, BD Symphony, BD Fortessa X50 or BD Aria II (BD Biosciences)
Software	FACS Diva Software version 8.0.1, FloJo v10.4.2
Cell population abundance	Sorted cells were run on an LSRII following collection. A purity of >90% was always observed.
Gating strategy	FSC-A/FSC-H and SCC-A/SSC-H plots were used to set the singlet gates. FSC-A/SCC-A plots were used to set cell population gates. Non-transduced cell populations were used to set sgRNA and shRNA gates. Isotype controls and unstained cells were used to define boundaries between populations.

- [x] Tick this box to confirm that a figure exemplifying the gating strategy is provided in the Supplementary Information.
