# Supplementary material for: N6-methyladenosine mRNA marking promotes selective translation of regulons required for human erythropoiesis
Source: Nat Commun. 2019 Oct 10;10:4596. doi: 10.1038/s41467-019-12518-6 (PMC6787028; doi:10.1038/s41467-019-12518-6)
Supplement: Supplementary file 3 — Description of Additional Supplementary Files [file 41467_2019_12518_MOESM3_ESM.docx]

**Description of Additional Supplementary Files**

File Name: Supplementary Data 1
Description: CRISPR-Cas9 CD235A^low^ enrichment screen data.

File Name: Supplementary Data 2
Description: MeRIP-seq analysis in HEL and adult human bone marrow HSPCs.

File Name: Supplementary Data 3
Description: RNA-seq analysis of *WTAP*, *METTL3, LMO2, GATA1 and GYPA* KO HEL cells. This data includes in separate tabs the complete RNAseq data, as well as GO analysis for the core groups of genes transcriptionally altered in both *METTL3*-KO and *WTAP*-KO samples.

File Name: Supplementary Data 4
Description: Splicing analysis of *METTL3*-KOand *WTAP*-KO HEL cells. This data includes in separate tabs the complete MISO and MATS splicing analyses.

File Name: Supplementary Data 5
Description: Ribosome profiling of *WTAP* KO HEL cells. This data includes in separate tabs the complete Riboseq data, as well as further analysis of the translationally down regulated genes and GO analysis. The data in the LogFC plot tab are presented in Fig. 4a. The data in the m^6^A translational down callouts tab presents all genes uniquely translationally down with known or suspected roles in hematopoietic progenitor cell function, erythropoiesis, anemia, and/or other hematopoietic diseases, of which a subset are presented in Fig. 4b. The GO tabs contain the ToppGene analysis of translationally up or down regulated genes in the context of being m^6^A methylated.

File Name: Supplementary Data 6
Description: CUT&RUN data. This data contains all called H3K4me3 peaks, gene list breakouts for the upset plot in Fig. 5c, analysis of enrichment for loss of H3K4me3 in erythropoiesis genes, and GO analysis of the non-KLF1 target genes with reduced H3K4me3.

File Name: Supplementary Data 7
Description: Erythroid Differentiation Gene Sets from Li, J., et al.

File Name: Supplementary Data 8
Description: sgRNAs, shRNAs, Luciferase reporter sequences and Primers used in this study.
